# Efficacy and safety of anti-CD38 monoclonal antibodies in patients with relapsed/refractory multiple myeloma: a systematic review and meta-analysis with trial sequential analysis of randomized controlled trials

**DOI:** 10.3389/fonc.2023.1240318

**Published:** 2023-12-07

**Authors:** Lu Ye, Fei Zhou, Dongdong Cheng, Ming Xie, Xiaoli Yan, Yuyu Xue, Qian Yang, Rong Jia, Lili Zhong, Li Yang, Liqun Zou, Na Huang

**Affiliations:** ^1^ Department of Medical Oncology of Cancer Center, West China Hospital, Sichuan University, Chengdu, China; ^2^ Department of Oncology, The Second Affiliated Hospital of Chengdu Medical College, China National Nuclear Corporation 416 Hospital, Chengdu, China; ^3^ Department of Obstetrics and Gynaecology, Sichuan Provincial People’s Hospital, University of Electronic Science and Technology of China, Chengdu, China; ^4^ Chinese Academy of Sciences Sichuan Translational Medicine Research Hospital, Chengdu, China; ^5^ Department of Publicity, The Third Hospital of Changsha, Changsha, China; ^6^ Department of Science and Education, The Third Hospital of Changsha, Changsha, China; ^7^ Department of Gynecology and Obstetrics, Southwest Hospital, Third Military Medical University (Army Medical University), Chongqing, China; ^8^ School of Preclinical Medicine, Chengdu University, Chengdu, China; ^9^ Clinical Medical College, Chengdu Medical College, Chengdu, China; ^10^ Department of Radiotherapy, Radiation Oncology Key Laboratory of Sichuan Province, Sichuan Clinical Research Center for Cancer, Sichuan Cancer Hospital & Institute, Sichuan Cancer Center, Affiliated Cancer Hospital of University of Electronic Science and Technology of China, Chengdu, China

**Keywords:** relapsed/refractory multiple myeloma, CD38, monoclonal antibodies, daratumumab, isatuximab, meta-analysis

## Abstract

**Objectives:**

The current study aims to evaluate the safety and efficacy of anti-CD38 monoclonal antibodies (mAbs) among patients with relapsed/refractory multiple myeloma (RRMM) through meta-analysis.

**Methods:**

As of June 2023, we searched PubMed, Web of Science, Embase and the Cochrane Library. Randomized controlled trials (RCTs) which compared the clinical outcomes of anti-CD38 mAbs plus immunomodulatory drugs (IMiDs) or proteasome inhibitors (PIs) plus dexamethasone and IMiDs (or PIs) and dexamethasone alone for RRMM patients were included. Efficacy outcomes were mainly evaluated with progression-free survival (PFS) and overall survival (OS). The safety was analyzed with hematologic and nonhematologic treatment-emergent adverse events (TEAEs). All results were pooled using hazard ratio (HR), relative risk (RR), and their 95% confidence interval (CI) and prediction interval (PI).

**Results:**

This meta-analysis included 11 RCTs in total. Compared with IMiDs (or PIs) and dexamethasone alone, anti-CD38 mAbs in combination with IMiDs (or PIs) and dexamethasone significantly prolonged PFS (HR: 0.552, 95% CI = 0.461 to 0.659, 95% PI = 0.318 to 0.957) and OS (HR: 0.737, 95% CI = 0.657 to 0.827, 95% PI = 0.626 to 0.868) in patients with RRMM. Additionally, RRMM patients receiving anti-CD38 mAbs in combination with IMiDs (or PIs) and dexamethasone achieved higher rates of overall response (RR: 1.281, 95% CI = 1.144 to 1.434, 95% PI = 0.883 to 1.859), complete response or better (RR: 2.602, 95% CI = 1.977 to 3.424, 95% PI = 1.203 to 5.628), very good partial response (VGPR) or better (RR: 1.886, 95% CI = 1.532 to 2.322, 95% PI = 0.953 to 3.731), and minimum residual disease (MRD)-negative (RR: 4.147, 95% CI = 2.588 to 6.644, 95% PI = 1.056 to 16.283) than those receiving IMiDs (or PIs) and dexamethasone alone. For TEAEs, the rates of hematologic and nonhematologic TEAEs, including thrombocytopenia, neutropenia, upper respiratory tract infection (URTI), pneumonia, bronchitis, dyspnea, diarrhea, pyrexia, back pain, arthralgia, fatigue, insomnia, and hypertension, were higher in the anti-CD38 mAbs in combination with IMiDs (or PIs) and dexamethasone group than in the IMiDs (or PIs) and dexamethasone group.

**Conclusion:**

Our study showed that anti-CD38 mAbs in combination with IMiDs (or PIs) and dexamethasone improved PFS and OS, and achieved higher rates of overall response, complete response or better, VGPR or better, and MRD-negative, as well as higher rates of thrombocytopenia, neutropenia, URTI, pneumonia, bronchitis, dyspnea, diarrhea, pyrexia, back pain, arthralgia, fatigue, insomnia, and hypertension in RRMM patients.

**Systematic review registration:**

https://www.crd.york.ac.uk/PROSPERO/, identifier CRD42023431071.

## 1 Introduction

Multiple myeloma (MM) is the second most common hematological malignancy in the world (1), with high morbidity and mortality (2). The development of novel treatments for MM has been conducive to enhanced survival and quality of life for patients (3). The immunomodulatory drugs (IMiDs) and proteasome inhibitors (PIs) have primarily driven the significant advancements in the therapeutic outcomes of MM patients over the past twenty years (4, 5). Shortly after these two drug classes established themselves as the cornerstone of MM treatment, a comprehensive study by the International Myeloma Working Group (IMWG) highlighted the grim prognosis for patients whose conditions exhibited resistance to both IMiDs and PIs. MM patients who received treatment before 2017 and were concurrently resistant to both an IMiD and a PI, had a median overall survival (OS) of 13 months, with a slim 33% probability of a positive response to the ensuing therapeutic line (6). Subsequent research revealed a variance in the median progression-free survival (PFS) and OS among MM patients receiving first-line treatment with IMiDs or PIs, depending on their International Staging System disease stage (7). Despite the improvements brought about by the introduction of several new drugs, almost all patients with MM ultimately become relapsed/refractory multiple myeloma (RRMM) (8). In order to address the unmet clinical requirements of RRMM patients, the spectrum of new agents has greatly expanded in the past decade (9, 10).

Monoclonal antibody (mAbs) targeting CD38 (e.g., daratumumab and isatuximab) have been generated because of the need for new approaches to treat RRMM (11, 12). Daratumumab is a human IgGκ mAb targeting CD38, which has direct effects on tumor (13, 14) and immune regulation mechanisms (15, 16), inducing induction of greater cytotoxicity of MM cells *in vitro* than other CD38 antibody analogues (17). Isatuximab is an IgG1 mAb targeting a unique epitope of CD38 (18). Isatuximab exerts its anti-myeloma effects through various mechanisms, such as direct induction of apoptotic cell death, complement-dependent cytotoxicity, and antibody-dependent cell-mediated cytotoxicity (19, 20). Previous study has reported the milestones of RRMM treatment included either a double-, triple-, or quadruple-drug combination on the basis of IMiDs and/or PIs and dexamethasone plus the anti-CD38 mAbs isatuximab or daratumumab, with or without chemotherapy (21).

Given the extensive research on anti-CD38 mAbs treatment for MM and the limited studies on the application of anti-CD38 mAbs in RRMM patients, there is an urgent need for healthcare stakeholders to compare the relative efficacy and safety of daratumumab or isatuximab versus standard therapy for RRMM. Several previous meta-analyses have reported the efficacy and safety of the triplet regimens containing daratumumab for treating MM (22–24). However, multiple related RCTs (e.g., POLLUX, CASTOR, CANDOR, and LEPUS) have subsequently updated their analysis results (25–28). Hence, we conducted a meta-analysis to systematically update the efficacy of anti-CD38 mAbs (daratumumab and isatuximab) for RRMM, and to supplement safety outcomes, including respiratory system treatment-emergent adverse events (TEAEs), digestive system TEAEs, and other nonhematologic TEAEs. Additionally, we performed subgroup analyses with the aim of providing new evidence for clinical practice.

## 2 Materials and methods

### 2.1 Study protocol

The reporting of this systematic review and meta-analysis complied with the Preferred Reporting Items for Systematic Reviews and Meta-analyses statement (PRISMA) (29). The protocol for our study was previously registered in the International Prospective Register of Systematic Reviews (PROSPERO CRD42023431071).

### 2.2 Search strategy

As of June 2023, we searched PubMed, Web of Science, Embase and the Cochrane Library using “daratumumab” or “humax-CD38” or “Darzalex” or “isatuximab” or “Sarclisa” or “SAR650984” or “isatuximab-irfc” or “SAR” or “multiple myeloma” or “plasma cell myeloma” or “myelomatosis” or “kahler disease” as MeSH terms and keywords. See **Supplementary Files 1** for the entire search strategy. For additional relevant literature, the authors also reviewed references in selected studies and reviews. Endnote X9 was used for further screening.

### 2.3 Inclusion and exclusion criteria

Clinical trials were included if they met the following criteria: (i) RCTs related to anti-CD38 mAbs (Daratumumab or Isatuximab) for treatment of RRMM; (ii) experimental arm: anti-CD38 mAbs in combination with IMiDs or PIs plus dexamethasone, control arm: IMiDs or PIs plus dexamethasone; (iii) for multiple articles published with the same trial, the latest study with the longest follow-up time or one that provided outcomes of interest was selected. The exclusion criteria were as follows: (i) retrospective or prospective cohort studies; (ii) clinical trials involving MM patients as participants; (iii) studies without relevant outcomes or with data duplication; (iv) conference abstracts, literature reviews, study protocols and case reports.

### 2.4 Data extraction and quality assessment

Endnote X9 was used to remove 1009 duplicate documents. Two investigators independently read the title/abstract and full text of the remaining articles, and extracted all needed information from the included studies. The conflicts between the two reviewers were resolved through discussion or the participation of a third reviewer. The collected information from each trial were as follows: first author name and publication year, region, sample size of participants, age of subjects, medication of the experimental and control groups, follow-up time, and trial name. PFS and OS were the primary outcomes; the other efficacy results and TEAEs were the secondary outcomes.

The modified Jadad scale was utilized for the quality and risk of bias of RCTs (30). An article with 0 to 3 scores considered to be of poor quality and that with 4 to 7 points suggested high quality. Two independent reviewers applied the modified Jadad scale and all disagreements can be resolved through consulting with a third investigator.

### 2.5 Statistical analysis

The data synthesis and analysis were performed by using R software 4.1.2 and STATA 12 (StataCorp, College Station, TX, USA). The efficacy and safety results are summarized using hazard ratio (HR) and relative risk (RR) with 95% confidence interval (CI) and prediction interval (PI). The overall study heterogeneity was assessed by the Cochran’s Q statistic and Higgin’s I^2^ test (31, 32). Acceptable heterogeneity was considered if the P >0.10 or I^2^ ≤ 50%. If no significant heterogeneity existed, we selected fixed-effect model; otherwise, the random-effect model was utilized (33). We conducted a sensitivity analysis through leave-one-out approach to find the possible sources of heterogeneity. Publication bias was tested and adjusted using funnel plots and the trim-and-fill method (34).

### 2.6 Trial sequential analysis

To further elicit the efficacy and safety of anti-CD38 mAbs in RRMM patients, we performed trial sequential analysis (TSA) using STATA 12 and R software 4.1.2, and TSA v0.9.5.10 (www.ctu.dk/tsa) for HR and RR, respectively. TSA was proposed to quantify the required sample information (RIS) and evaluate whether a robust conclusion can be drawn based on the available evidence (35). The “metacumbounds” and “rsource” function of STATA 12, together with the “foreign” and “ldbounds” packages of R software were used for the efficacy results of PFS and OS. The RIS was assessed by an *a priori* information size (APIS) method. We used TAS software to estimate the RIS and build O’ Brien-Fleming α-spending boundariesfor the pooled effect value of RR, considering type I error of 5% and type II error of 20%. Statistical significance was set at P < 0.05.

## 3 Results

### 3.1 Literature search

**Figure 1:**
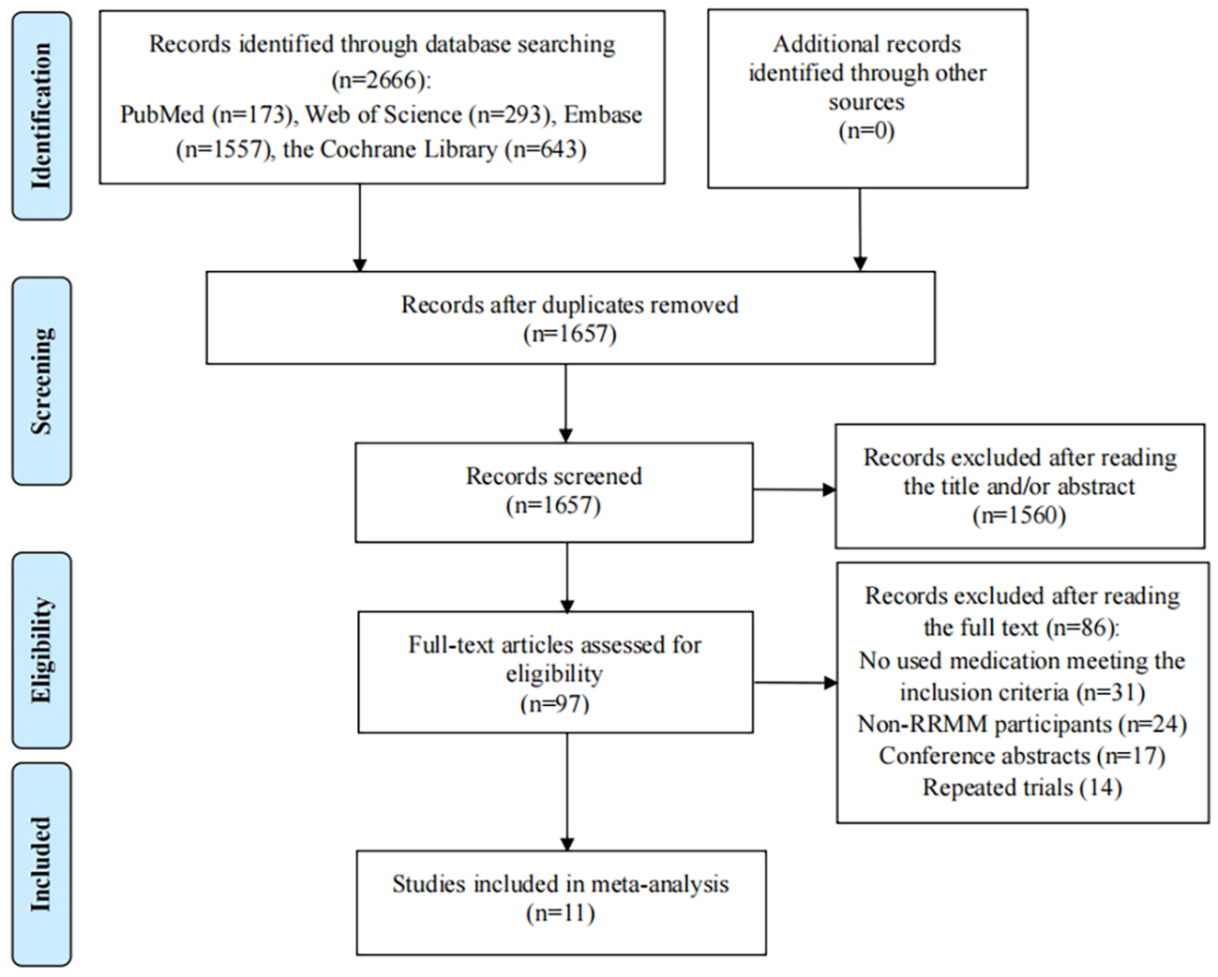
Flow diagram of the studies included in the meta-analysis.

The study selection is described in **Figure 1**. A total of 2666 references were retrieved from PubMed (n=173), Web of Science (n=293), Embase (n=1557) and the Cochrane Library (n=643). After 1009 duplicate literature were removed, the remaining 1657 articles were screened. The titles/abstracts of the remaining 1657 articles were reviewed and 1560 documents were excluded because their titles/abstracts were not relevant. After screening 97 full texts, 86 articles were excluded: the medication used in the experimental and control groups reported in 31 studies did not follow the inclusion criteria; the participants in 24 studies were not patients with RRMM; 17 literatures were conference abstracts; 14 articles reported repeated trials. Finally, 11 RCTs were included in the meta-analysis (25–28, 36–42).

### 3.2 Characteristics and quality assessment of the included studies

**Table 1** describes the characteristics of included research. The included 11 studies were prospective, randomized, open-label, multicentre, phase 3 clinical trials. For the repeated trial (including CANDOR, CASTOR, LEPUS, IKEMA and POLLUX), although it has been analyzed and reported in multiple articles, we included the latest studies with longest follow-up time (e.g., Usmani et al, 2023) and studies that provided outcomes of interest (e.g., Usmani et al, 2022). Patients with RRMM who had received ≥ 1 prior lines of treatment were eligible. Patients were randomly assigned to anti-CD38 mAbs (Daratumumab or Isatuximab) plus IMiDs (or PIs) and dexamethasone (experimental group) or IMiDs (or PIs) and dexamethasone alone (control group). 9 included RCTs were assessed as high quality, because the study design had been detailly described. The remaining two studies were evaluated as low quality, as randomization, randomization concealment, and withdrawals and dropouts were not described in detail (**Supplementary Files 2**).

**Table 1:** Characteristics of the studies included in the meta-analysis.

First author (year)	Region	Participants (E/C)	Age (E/C, years)	Intervention (E/C)	Previous lines of treatment	Follow-up time (months)	Trial
Richardson (2022)	102 hospitals in 24 countries across Europe, North America, and the Asia-Pacific regions	154/153	Median (IQR): 68 (60–74)/66 (59-71)	Isatuximab + pomalidomide + dexamethasone/Pomalidomide + dexamethasone	≥ 2	35.3 (median)	ICARIA-MM
Usmani (2023)	102 medical centers in 19 countries across North America, Europe, Australia, and Asia	312/154	Median (IQR): 64.0 (57-70)/64.5 (59-71)	Daratumumab + carfilzomib + dexamethasone/Carfilzomib + dexamethasone	1-3	50 (median)	CANDOR
Sonneveld (2023)	16 countries across Europe, North America, South America, Australia, and Asia	251/247	Median (range): 64 (30-88)/64 (33-85)	Daratumumab + bortezomib + dexamethasone/Bortezomib + dexamethasone	≥ 1	72.6 (median)	CASTOR
Lu (2021)	Mainland China (25 sites) and Taiwan (2 sites)	141/70	Median (range): 61 (28-79)/61 (43-82)	Daratumumab + bortezomib + dexamethasone/Bortezomib + dexamethasone	≥ 1	8.2 (median)	LEPUS
Martin (2023)	69 study centers in 16 countries across North America, South America, Europe, and the Asia-Pacific region.	179/123	Median (range): 65 (37-86)/63 (33-90)	Isatuximab + carfilzomib + dexamethasone/Carfilzomib + dexamethasone	1-3	44 (median)	IKEMA
Dimopoulos (2021)	48 academic centers and hospitals in 12 European countries	151/153	Median (range): 67 (42-86)/68 (35-90)	Daratumumab + pomalidomide + dexamethasone/Pomalidomide + dexamethasone	≥ 1	16.9 (median)	APOLLO
Bahlis (2020)	135 sites in 18countries across North America, Europe, and the Asia Pacific region	286/283	Median (range): 65 (34-89)/65 (42-87)	Daratumumab + lenalidomide + dexamethasone/Lenalidomide + dexamethasone	≥ 1	44.3 (median)	POLLUX
Fu (2023)	Mainland China (25 sites) and Taiwan (2 sites)	141/70	Median (range): 61 (28-79)/61 (43-82)	Daratumumab + bortezomib + dexamethasone/Bortezomib + dexamethasone	≥ 1	25.1 (median)	LEPUS
Usmani (2022)	102 medical centers in 19 countries across North America, Europe, Australia, and Asia	312/154	Median (IQR): 64.0 (57-70)/64.5 (59-71)	Daratumumab + carfilzomib + dexamethasone/Carfilzomib + dexamethasone	1-3	27	CANDOR
Dimopoulos (2023)	135 sites in 18countries across North America, Europe, and the Asia Pacific region	286/283	Median (range): 65 (34-89)/65 (42-87)	Daratumumab + lenalidomide + dexamethasone/Lenalidomide + dexamethasone	≥ 1	79.7 (median)	POLLUX
Mateos (2020)	16 countries across Europe, North America, South America, Australia, and Asia	251/247	Median (range): 64 (30-88)/64 (33-85)	Daratumumab + bortezomib + dexamethasone/Bortezomib + dexamethasone	≥ 1	40 (median)	CASTOR

E, experimental arm; C, control arm; IQR, interquartile range.

### 3.3 Pooled effect of efficacy outcomes

**Figure 2:**
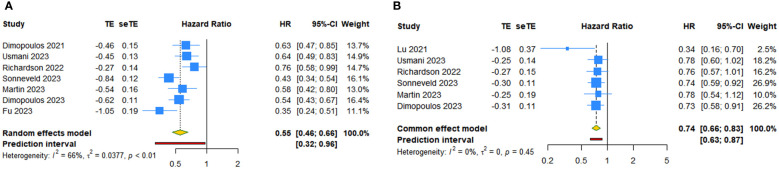
Forest plot of progression-free survival (PFS) and overall survival (OS) after anti-CD38 mAbs therapy for RRMM. **(A)** PFS; **(B)** OS.

Seven RCTs examined the PFS benefit of anti-CD38 mAbs in patients with RRMM. The random-effects pooled estimate showed that anti-CD38 mAbs in combination with IMiDs (or PIs) and dexamethasone resulted in a 44.8% reduction in the risk of disease progression or death compared with IMiDs (or PIs) and dexamethasone alone (HR: 0.552, 95% CI = 0.461 to 0.659, 95% PI = 0.318 to 0.957; I^2 ^= 66.1%, Tau^2 ^= 0.0377) (**Table 2**, **Figure 2A**). Subgroup analysis revealed that daratumumab (HR: 0.513, 95% CI = 0.420 to 0.626, 95% PI = 0.266 to 0.989; I^2 ^= 63.4%, Tau^2 ^= 0.0323) or isatuximab (HR: 0.679, 95% CI = 0.554 to 0.833; I^2 ^= 39.0%, Tau^2 ^= 0.0142) significantly prolonged PFS (**Table 2**; **Supplementary Files 3**, **Supplementary Figure 1A**). OS benefit was evaluated in six studies. The overall results showed that anti-CD38 mAbs significantly prolonged OS in patients with RRMM (HR: 0.737, 95% CI = 0.657 to 0.827, 95% PI = 0.626 to 0.868; I^2 ^= 0%, Tau^2 ^= 0) (**Table 2**, **Figure 2B**). Subgroup analysis suggested that daratumumab (HR: 0.726, 95% CI = 0.635 to 0.831, 95% PI = 0.399 to 1.285; I^2 ^= 33.7%, Tau^2 ^= 0.0105) or isatuximab (HR: 0.768, 95% CI = 0.613 to 0.961; I^2 ^= 0, Tau^2 ^= 0) significantly prolonged OS (**Table 2**; **Supplementary Files 3**, **Supplementary Figure 1B**).

**Table 2:** Summary risk estimates for the efficacy outcomes of anti-CD38 mAbs for RRMM.

Outcomes and groups	Number of study	Meta-analysis				Heterogeneity	
		RR/HR	95% CI	P value	95% PI	I2, Tau2	P value
PFS	7	0.552	0.461-0.659	<0.001	0.318-0.957	66.1%, 0.0377	0.007
Daratumumab	5	0.513	0.420-0.626	<0.001	0.266-0.989	63.4%, 0.0323	0.027
Isatuximab	2	0.679	0.554-0.833	<0.001	–	39.0%, 0.0142	0.201
OS	6	0.737	0.657-0.827	<0.001	0.626-0.868	0%, 0	0.453
Daratumumab	4	0.726	0.635-0.831	<0.001	0.399-1.285	33.7%, 0.0105	0.210
Isatuximab	2	0.768	0.613-0.961	0.021	–	0%, 0	0.913
Overall response rate	7	1.281	1.144-1.434	<0.001	0.883-1.859	82.3%, 0.0177	<0.001
Daratumumab	5	1.265	1.201-1.333	<0.001	1.025-1.549	43.5%, 0.0028	0.132
Isatuximab	2	1.387	0.672-2.862	0.376	–	96.5%, 0.2639	<0.001
Complete response or better rate	7	2.602	1.977-3.424	<0.001	1.203-5.628	58.6%, 0.0705	0.025
Daratumumab	5	2.773	2.319-3.316	<0.001	1.649-4.666	21.7%, 0.0141	0.277
Isatuximab	2	2.064	0.911-4.677	0.083	–	58.2%, 0.2310	0.122
VGPR or better rate	7	1.886	1.532-2.322	<0.001	0.953-3.731	82.2%, 0.0592	<0.001
Daratumumab	5	1.869	1.544-2.262	<0.001	0.978-3.569	72.2%, 0.0319	0.006
Isatuximab	2	2.128	0.699-6.472	0.184	–	94.2%, 0.6082	<0.001
MRD-negative rate	6	4.147	2.588-6.644	<0.001	1.056-16.283	59.8%, 0.1849	0.029
Daratumumab	5	4.882	3.529-6.753	<0.001	1.990-11.731	19.1%, 0.0384	0.293
Isatuximab	1	2.170	1.367-3.445	0.001			

PFS, progression-free survival; OS, overall survival; VGPR, very good partial response; MRD, minimum residual disease.

–, not available.

Subgroup analysis of PFS revealed that the combination of daratumumab with IMiDs (or PIs) and dexamethasone significantly extended PFS when subgrouped by sex, age, International Staging System disease staging, type of measurable multiple myeloma, number of prior lines of therapy, or cytogenetic profile (all P < 0.05) (**Supplementary Files 4**, **Supplementary Table 1**, **Supplementary Figure 1**). However, we found no association between the addition of daratumumab to IMiDs (or PIs) and dexamethasone and prolonged OS in MM patients with non-IgG, or RRMM patients with 2, 3 or >3 prior lines of therapy (all P > 0.05) (**Supplementary Files 4**, **Supplementary Table 2**, **Supplementary Figure 2**).

**Figure 3:**
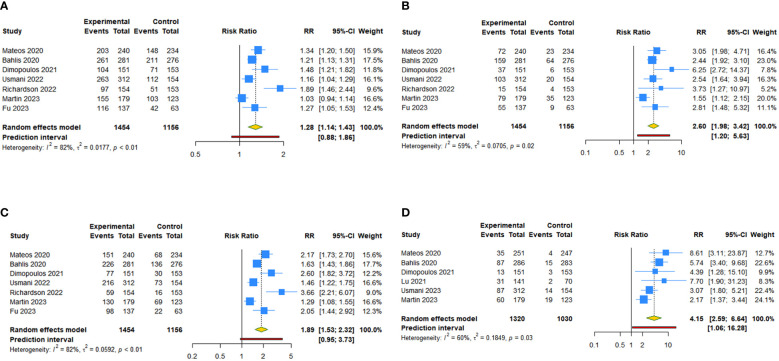
Forest plot of the efficacy outcomes after anti-CD38 mAbs therapy for RRMM. **(A)** Overall response rate; **(B)** Complete response or better rate; **(C)** Very good partial response or better rate; **(D)** Minimum residual disease-negative rate.

There were seven studies focused on the outcomes of overall response rate, complete response or better rate, and very good partial response (VGPR) or better rate. The results showed that mAbs achieved higher overall response rate (RR: 1.281, 95% CI = 1.144 to 1.434, 95% PI = 0.883 to 1.859; I^2 ^= 82.3%, Tau^2 ^= 0.0177), complete response or better rate (RR: 2.602, 95% CI = 1.977 to 3.424, 95% PI = 1.203 to 5.628; I^2 ^= 58.6%, Tau^2 ^= 0.0705), and VGPR or better rate (RR: 1.886, 95% CI = 1.532 to 2.322, 95% PI = 0.953 to 3.731; I^2 ^= 82.2%, Tau^2 ^= 0.0592) (**Table 2**, **Figures 3A-C**). Compared with the control groups, daratumumab plus IMiDs (or PIs) and dexamethasone significantly improved overall response rate (RR: 1.265, 95% CI = 1.201 to 1.333, 95% PI: 1.025 to 1.549; I^2 ^= 43.5%, Tau^2 ^= 0.0028), complete response or better rate (RR: 2.773, 95% CI = 2.319 to 3.316, 95% PI = 1.649 to 4.666; I^2 ^= 21.7%, Tau^2 ^= 0.0141), and VGPR or better rate (RR: 1.869, 95% CI = 1.544 to 2.262, 95% PI = 0.978 to 3.569; I^2 ^= 72.2%, Tau^2 ^= 0.0319). Isatuximab plus IMiDs (or PIs) and dexamethasone seem to achieved higher overall response rate, complete response or better rate and VGPR or better rate, but without statistical significance (all P>0.05) (**Table 2**; **Supplementary Files 3**, **Supplementary Figures 2A–C**).

There were six studies focused on minimum residual disease (MRD)-negative rate. Compared with the control groups, anti-CD38 mAbs improved MRD-negative rate (RR: 4.147, 95% CI = 2.588 to 6.644, 95% PI = 1.056 to 16.283; I^2 ^= 59.8%, Tau^2 ^= 0.1849) (**Table 2**, **Figure 3D**). Daratumumab plus IMiDs (or PIs) and dexamethasone significantly achieved higher MRD-negative rate (RR: 4.882, 95% CI = 3.529 to 6.753, 95% PI = 1.990 to 11.731; I^2 ^= 19.1%, Tau^2 ^= 0.0384). Isatuximab for patients with RRMM did not achieve higher MRD-negative rate (P > 0.05) (**Table 2**; **Supplementary Files 3**, **Supplementary Figure 2D**).

### 3.4 Pooled effect of safety outcomes

#### 3.4.1 Hematologic TEAEs

**Figure 4:**
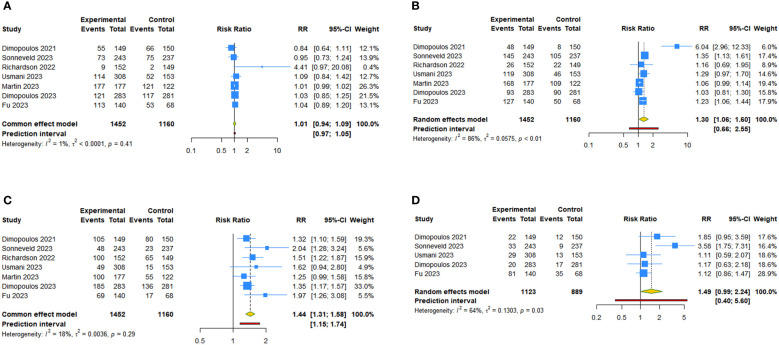
Forest plot of the hematologic treatment-emergent adverse events of anti-CD38 mAbs therapy for RRMM. **(A)** Anemia; **(B)** Thrombocytopenia; **(C)** Neutropenia; **(D)** Lymphopenia.

Seven studies assessed anemia, thrombocytopenia and neutropenia after anti-CD38 mAbs therapy for RRMM. The overall analysis showed that anti-CD38 mAbs increased the risks of thrombocytopenia (RR: 1.301, 95% CI = 1.055 to 1.603, 95% PI = 0.663 to 2.554; I^2 ^= 86.4%, Tau^2 ^= 0.0575) and neutropenia (RR: 1.441, 95% CI = 1.315 to 1.579, 95% PI = 1.154 to 1.740; I^2 ^= 18.5%, Tau^2 ^= 0.0036) compared with the control (**Table 3**, **Figures 4A-C**). The subgroup analysis revealed that daratumumab plus IMiDs (or PIs) and dexamethasone significantly increased the incidence of thrombocytopenia (RR: 1.405, 95% CI = 1.084 to 1.821, 95% PI = 0.563 to 3.507; I^2 ^= 82.7%, Tau^2 ^= 0.0651) and neutropenia (RR: 1.469, 95% CI = 1.313 to 1.642, 95% PI = 0.970 to 2.230; I^2 ^= 35.9%, Tau^2 ^= 0.0107), and isatuximab increased the risk of neutropenia (RR: 1.381, 95% CI = 1.179 to 1.619; I^2 ^= 23.0%, Tau^2 ^= 0.0039) compared with the control. No statistically significant results were obtained for anemia after daratumumab and isatuximab treatment in patients with RRMM (all P>0.05) (**Table 3**; **Supplementary Files 3**, **Supplementary Figures 3A–C**). There were five studies focused on lymphopenia after daratumumab therapy for RRMM. The pooled RR was 1.489 (95% CI = 0.991 to 2.237, 95% PI = 0.396 to 5.603; I^2 ^= 63.5%, Tau^2 ^= 0.1303), indicating daratumumab seem to increase lymphopenia risk, but without statistical significance (P > 0.05) (**Table 3**, **Figure 4D**).

**Table 3:** Summary risk estimates for the hematologic TEAEs of anti-CD38 mAbs for RRMM.

Outcomes and groups	Number of study	Meta-analysis				Heterogeneity	
		RR	95% CI	P value	95% PI	I^2^, Tau^2^	P value
Anemia	7	1.010	0.937-1.090	0.789	0.968-1.050	1.4%, <0.0001	0.414
Daratumumab	5	0.994	0.896-1.101	0.903	0.862-1.170	0%, 0	0.661
Isatuximab	2	2.066	0.022-196.625	0.755	–	97.2%, 10.514	<0.001
Thrombocytopenia	7	1.301	1.055-1.603	0.014	0.663-2.554	86.4%, 0.0575	<0.001
Daratumumab	5	1.405	1.084-1.821	0.010	0.563-3.507	82.7%, 0.0651	<0.001
Isatuximab	2	1.077	0.975-1.189	0.145	–	0%, 0	0.645
Neutropenia	7	1.441	1.315-1.579	<0.001	1.154-1.740	18.5%, 0.0036	0.289
Daratumumab	5	1.469	1.313-1.642	<0.001	0.970-2.230	35.9%, 0.0107	0.182
Isatuximab	2	1.381	1.179-1.619	<0.001	–	23.0%, 0.0039	0.255
Lymphopenia	5	1.489	0.991-2.237	0.055	0.396-5.603	63.5%, 0.1303	0.027
Daratumumab	5	1.489	0.991-2.237	0.055	0.396-5.603	63.5%, 0.1303	0.027

–, not available.

#### 3.4.2 Nonhematologic TEAEs

**Figure 5:**
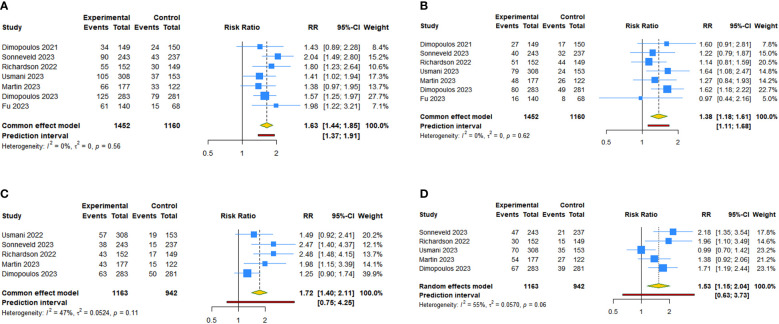
Forest plot of respiratory system treatment-emergent adverse events of anti-CD38 mAbs therapy for RRMM. **(A)** Upper respiratory tract infection; **(B)** Pneumonia; **(C)** Bronchitis; **(D)** Dyspnea.z.

There were seven studies and five studies, focused on TEAEs of respiratory system, respectively. The result showed that compared with the control, anti-CD38 mAbs achieved higher incidence of upper respiratory tract infection (URTI) (RR: 1.629, 95% CI = 1.436 to 1.848, 95% PI = 1.373 to 1.912; I^2 ^= 0%, Tau^2 ^= 0), pneumonia (RR: 1.380, 95% CI = 1.180 to 1.614, 95% PI = 1.114 to 1.680; I^2 ^= 0%, Tau^2 ^= 0), bronchitis (RR: 1.717, 95% CI = 1.400 to 2.106, 95% PI = 0.746 to 4.254; I^2 ^= 47.4%, Tau^2 ^= 0.0524) and dyspnea (RR: 1.533, 95% CI = 1.155 to 2.036, 95% PI = 0.630 to 3.729; I^2 ^= 55.4%, Tau^2 ^= 0.0570) (**Table 4**, **Figure 5**). Daratumumab significantly increased the risks of URTI (RR: 1.651, 95% CI = 1.428 to 1.908, 95% PI = 1.298 to 2.077; I^2 ^= 0%, Tau^2 ^= 0), pneumonia (RR: 1.480, 95% CI = 1.217 to 1.799, 95% PI = 1.078 to 2.036; I^2 ^= 0%, Tau^2 ^= 0), and bronchitis (RR: 1.576, 95% CI = 1.084 to 2.292, 95% PI = 0.033 to 76.027; I^2 ^= 51.4%, Tau^2 ^= 0.0565). Isatuximab plus IMiDs (or PIs) and dexamethasone achieved higher incidence of URTI (RR: 1.561, 95% CI = 1.207 to 2.020; I^2 ^= 0.8%, Tau^2 ^= 0.0003), bronchitis (RR: 2.223, 95% CI = 1.533 to 3.225; I^2 ^= 0%, Tau^2 ^= 0), and dyspnea (RR: 1.566, 95% CI = 1.127 to 2.175; I^2 ^= 0%, Tau^2 ^= 0) (**Table 4**; **Supplementary Files 3**, **Supplementary Figure 4**).

**Table 4:** Summary risk estimates for the nonhematologic TEAEs of anti-CD38 mAbs for RRMM.

Outcomes and groups	Number of study	Meta-analysis				Heterogeneity	
		RR	95% CI	P value	95% PI	I^2^, Tau^2^	P value
URTI	7	1.629	1.436-1.848	<0.001	1.373-1.912	0%, 0	0.559
Daratumumab	5	1.651	1.428-1.908	<0.001	1.298-2.077	0%, 0	0.442
Isatuximab	2	1.561	1.207-2.020	<0.001	–	0.8%, 0.0003	0.315
Pneumonia	7	1.380	1.180-1.614	<0.001	1.114-1.680	0%, 0	0.620
Daratumumab	5	1.480	1.217-1.799	<0.001	1.078-2.036	0%, 0	0.650
Isatuximab	2	1.192	0.918-1.548	0.188	–	0%, 0	0.677
Bronchitis	5	1.717	1.400-2.106	<0.001	0.746-4.254	47.4%, 0.0524	0.107
Daratumumab	3	1.576	1.084-2.292	0.017	0.033-76.027	51.4%, 0.0565	0.128
Isatuximab	2	2.223	1.533-3.225	<0.001	–	0%, 0	0.551
Dyspnea	5	1.533	1.155-2.036	0.003	0.630-3.729	55.4%, 0.0570	0.062
Daratumumab	3	1.518	0.964-2.391	0.072	0.008-300.972	74.7%, 0.1196	0.019
Isatuximab	2	1.566	1.127-2.175	0.008	–	0%, 0	0.324
Diarrhea	7	1.535	1.370-1.721	<0.001	1.201-1.908	13.4%, 0.0040	0.328
Daratumumab	5	1.607	1.412-1.829	<0.001	1.142-2.201	16.2%, 0.0049	0.311
Isatuximab	2	1.312	1.030-1.671	0.028	–	0%, 0	0.595
Constipation	4	1.050	0.776-1.422	0.751	0.313-3.521	59.1%, 0.0551	0.062
Daratumumab	3	1.120	0.791-1.586	0.522	0.025-50.936	63.1%, 0.0588	0.067
Isatuximab	1	0.817	0.505-1.321	0.409			
Pyrexia	6	1.504	1.258-1.799	<0.001	1.166-1.938	0%, 0	0.666
Daratumumab	5	1.540	1.273-1.863	<0.001	1.129-2.098	0%, 0	0.605
Isatuximab	1	1.260	0.747-2.128	0.386			
Back pain	5	1.407	1.177-1.682	<0.001	0.786-2.480	31.2%, 0.0198	0.213
Daratumumab	3	1.541	1.241-1.913	<0.001	0.069-35.558	46.6%, 0.0353	0.154
Isatuximab	2	1.147	0.835-1.576	0.397	–	0%, 0	0.789
Arthralgia	4	1.692	1.066-2.686	0.026	0.236-12.113	70.9%, 0.1537	0.016
Daratumumab	2	2.066	0.812-5.256	0.128	–	88.2%, 0.4020	0.004
Isatuximab	2	1.435	0.948-2.171	0.088	–	36.7%, 0.0541	0.209
Fatigue	6	1.213	1.060-1.388	0.005	0.853-1.702	22.8%, 0.0089	0.263
Daratumumab	4	1.198	1.030-1.394	0.019	0.623-2.251	32.8%, 0.0127	0.216
Isatuximab	2	1.266	0.942-1.701	0.118	–	48.6%, 0.0436	0.163
Asthenia	5	1.069	0.880-1.298	0.504	0.551-2.056	34.6%, 0.0271	0.191
Daratumumab	3	1.082	0.740-1.582	0.686	0.018-64.575	58.4%, 0.0659	0.090
Isatuximab	2	1.021	0.726-1.436	0.904	–	14.6%, 0.0105	0.279
Insomnia	5	1.292	1.080-1.545	0.005	0.932-1.729	1.9%, 0.0009	0.395
Daratumumab	4	1.358	1.112-1.659	0.003	0.861-2.069	0%, 0	0.421
Isatuximab	1	1.034	0.693-1.543	0.870			
Hypertension	4	1.722	1.027-2.887	0.040	0.189-15.690	79.7%, 0.1942	0.002
Daratumumab	3	2.373	0.907-6.205	0.078	–	84.4%, 0.5921	0.002
Isatuximab	1	1.074	0.791-1.458	0.647			

URTI, upper respiratory tract infection.

–, not available.

**Figure 6:**
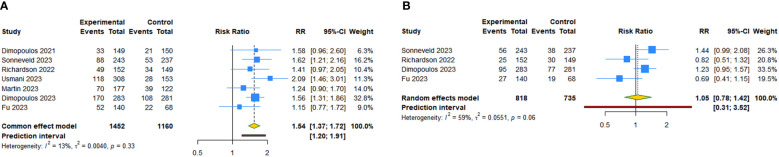
Forest plot of digestive system treatment-emergent adverse events of anti-CD38 mAbs therapy for RRMM. **(A)** Diarrhea; **(B)** Constipation.

Seven studies and four studies reported TEAEs of digestive system, including diarrhea and constipation. The overall and subgroup analysis showed that anti-CD38 mAbs (RR: 1.535, 95% CI = 1.370 to 1.721, 95% PI = 1.201 to 1.908; I^2 ^= 13.4%, Tau^2 ^= 0.0040) (**Table 4**, **Figure 6A**), daratumumab (RR: 1.607, 95% CI = 1.412 to 1.829, 95% PI = 1.142 to 2.201; I^2 ^= 16.2%, Tau^2 ^= 0.0049) or isatuximab (RR: 1.312, 95% CI = 1.030 to 1.671; I^2 ^= 0%, Tau^2 ^= 0) increased the diarrhea risk (**Table 4**; **Supplementary Files 3**, **Supplementary Figure 5A**). No statistically significant results were obtained for constipation after daratumumab and isatuximab therapy (all P>0.05) (**Table 4**; **Figure 6B**, **Supplementary Files 3**, **Supplementary Figure 5B**).

**Figure 7:**
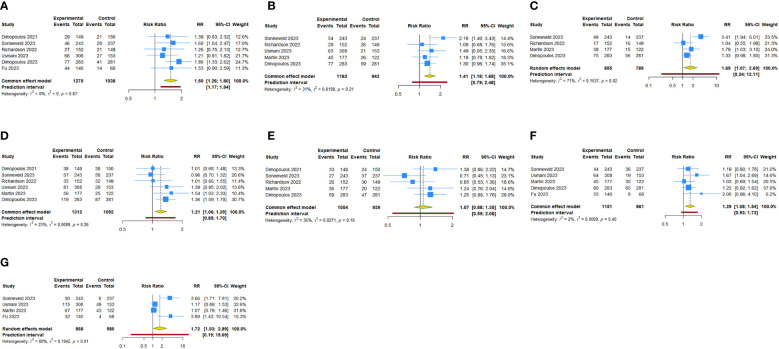
Forest plot of other nonhematologic treatment-emergent adverse events of anti-CD38 mAbs therapy for RRMM. **(A)** Pyrexia; **(B)** Back pain; **(C)** Arthralgia; **(D)** Fatigue; **(E)** Asthenia; **(F)** Insomnia; **(G)** Hypertension.

For other nonhematologic TEAEs, the overall analysis showed that compared with the control groups, mAbs plus IMiDs (or PIs) and dexamethasone significantly increased the risks of pyrexia (RR: 1.504, 95% CI = 1.258 to 1.799, 95% PI = 1.166 to 1.938; I^2 ^= 0%, Tau^2 ^= 0), back pain (RR: 1.407, 95% CI = 1.177 to 1.682, 95% PI = 0.786 to 2.480; I^2 ^= 31.2%, Tau^2 ^= 0.0198), arthralgia (RR: 1.692, 95% CI = 1.066 to 2.686, 95% PI = 0.236 to 12.113; I^2 ^= 70.9%, Tau^2 ^= 0.1537), fatigue (RR: 1.213, 95% CI = 1.060 to 1.388, 95% PI = 0.853 to 1.702; I^2 ^= 22.8%, Tau^2 ^= 0.0089), insomnia (RR: 1.292, 95% CI = 1.080 to 1.545, 95% PI = 0.932 to 1.729; I^2 ^= 1.9%, Tau^2 ^= 0.0009), and hypertension (RR: 1.722, 95% CI = 1.027 to 2.887, 95% PI = 0.189 to 15.690; I^2 ^= 79.7%, Tau^2 ^= 0.1942) (**Table 4**, **Figure 7**). The subgroup analysis revealed that daratumumab plus IMiDs (or PIs) and dexamethasone achieved higher incidence of pyrexia (RR: 1.540, 95% CI = 1.273 to 1.863, 95% PI = 1.129 to 2.098; I^2 ^= 0%, Tau^2 ^= 0), back pain (RR: 1.541, 95% CI = 1.241 to 1.913, 95% PI = 0.069 to 35.558; I^2 ^= 46.6%, Tau^2 ^= 0.0353), fatigue (RR: 1.198, 95% CI = 1.030 to 1.394, 95% PI = 0.623 to 2.251; I^2 ^= 32.8%, Tau^2 ^= 0.0127), and insomnia (RR: 1.358, 95% CI = 1.112 to 1.659, 95% PI = 0.861 to 2.069; I^2 ^= 0%, Tau^2 ^= 0). No statistically significant results were observed for pyrexia, back pain, arthralgia, fatigue, asthenia, insomnia, and hypertension after isatuximab therapy in patients with RRMM (all P > 0.05) (**Table 4**; **Supplementary Files 3**, **Supplementary Figure 6**).

### 3.5 TSA results

**Figure 8:**
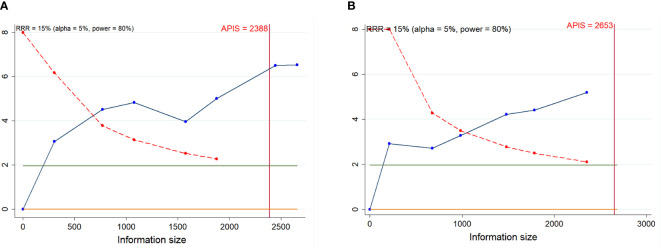
Trial sequential analysis (TSA) of progression-free survival (PFS) and overall survival (OS) after anti-CD38 mAbs therapy for RRMM. **(A)** PFS; **(B)** OS. Red inward-sloping line to the left represents trial sequential monitoring boundary. Blue line represents evolution of cumulative Z-score. Horizontal green lines represent the conventional boundaries for statistical significance. Heterogeneity-adjusted required information size to demonstrate or reject 15% relative risk (*a priori* estimate) of mortality risk (with alpha of 5% and beta of 20%) is 2388 patients for PFS and 2653 patients for OS (vertical red line). Cumulative Z-curve crossing the trial sequential monitoring boundary or the APIS boundary provides firm evidence of effect.

We estimated a RIS of 2388 and 2653 for PFS and OS, respectively. Both cumulative Z-curves successfully passed the trial sequential monitoring boundary, but merely the cumulative Z-curve of PFS crossed the RIS boundary, which suggested that an adequate level of evidence was reached for PFS and OS (**Figure 8**). For other efficacy outcomes, the cumulative Z-curves crossed both the RIS boundary and trial sequential monitoring boundary, indicating that a relatively definite conclusion can be obtained (**Supplementary Files 5**, **Supplementary Figure 1**). For TEAEs, only the cumulative Z-curves of anemia, thrombocytopenia, lymphopenia, constipation, arthralgia, asthenia and hypertension did not cross either the trial sequential monitoring boundary or RIS boundary, and thus we cannot draw a robust conclusion about these TEAEs due to the presence of false positive (**Supplementary Files 5**, **Supplementary Figures 2–5)**.

### 3.6 Publication bias and sensitivity analysis

The publication bias tests and sensitivity analyses were performed merely for the efficacy and safety results which included ≥ 7 studies. Trim-and-fill technique was applied to suggest and adjust for publication bias. We found funnel plot region contained missing studies in the outcomes of overall response rate, complete response or better rate, VGPR or better rate, anemia, thrombocytopenia and neutropenia, indicating that publication bias was significant. After adjusting for publication bias, the adjusted results are consistent with the previous results, suggesting that these results are still credible. It has been presented in **Supplementary Files 6**, **Supplementary Figures 1–3**.

We performed sensitivity analyses through the leave-one-out approach to further test the stability of the results. The results suggested that Richardson et al.’s article might be the cause of large heterogeneity of the outcomes of overall response rate and VGPR or better rate; Dimopoulos et al. (2021).’s research is possibly the source of high heterogeneity of the outcome of thrombocytopenia (**Supplementary Figures 4–6**, **Supplementary Files 6**).

## 4 Discussion

MM is a plasma cell malignant disorder characterized by uncontrollable and progressive proliferation of plasma cell clones, leading to the excessive production of nonfunctional intact immunoglobulins or immunoglobulin chains (43). The accumulation of these immunoglobulins and the interaction between abnormal monoclonal plasma cells and other cells in bone marrow result in many AEs including bone injury, renal failure, hypercalcemia, infections, anemia, pain and fatigue (44). In the early 2000s, the introduction of IMiDs and PIs altered the outcomes of MM patients. Later, detecting CD38 as appropriate target resulted in immunotherapy methods using mAbs such as daratumumab and isatuximab (45). Although people have gained a better recognition of the biology of the disorder and introduced treatment options with new mechanisms, current treatments cannot cure MM. The majority of patients still experience disease relapse and frequently exhibit tolerance to previously used medications (46). RRMM is defined as a disorder that does not respond or progresses in patients who have had the least or better response to previous treatment within 60 days after the last treatment or the last treatment (47). Due to the poor therapy prognosis, drug resistance, and severe impairment of patients’ quality of life, RRMM has become a major problem currently faced by clinical doctors (48–50). The insignificant treatment effects of prior medications have promoted the development of the next generation of PIs and IMiDs, as well as regimen with anti-CD38 mAbs, further expanding the therapeutic range of RRMM (51, 52). Therefore, the development of novel therapeutic drugs targeting CD38 has become a hot direction for the treatment of RRMM.

CD38 is a type II transmembrane protein, which is expressed in immune cells and regulates calcium signaling, leukocyte activation and migration process (45). CD38 is expressed at a low level in immune cells under normal circumstances (53), while obviously higher CD38 expression in plasma cells is shown in healthy individuals and MM patients (20). Daratumumab binds to transmembrane glycoprotein CD38 with high affinity (54) and inhibits tumor cell growth through immunemediated direct on-tumor (55) and immunomodulatory mechanisms of action (15). It has been approved by the European Medicines Agency (EMA) and US Food and Drug Administration (FDA) as both single drug therapy and combined with standard treatment for RRMM, following intravenous and SC subcutaneous. Isatuximab is a novel IgG1 monoclonal antibody, which can directly regulate the enzyme activity of CD38, induce MM cell death by caspase-dependent apoptosis, and improve the immune response mediated by T cells and natural killer cells (20). Despite the similar action of daratumumab and isatuximab, the mechanism of both anti-CD38 mAbs is somewhat different. On one side, daratumumab and isatuximab target different CD38 epitopes. Additionally, isatuximab can directly induce cell death, whereas daratumumab must be in combination with cross-linking agents to induce cell apoptosis (20, 53).

Daratumumab has been used as a single drug in clinical practice and in combination with PIs (i.e., carfilzomib and bortezomib) or IMiDs (i.e., pomalidomide and lenalidomide) and demonstrated activity in patients with RRMM (56–59). All RRMM patients in this meta-analysis had received ≥ 1 lines of therapy and gained clinical benefits from daratumumab plus IMiDs or PIs plus dexamethasone. In present analysis, the addition of daratumumab to IMiDs (or PIs) and dexamethasone significantly improved PFS and OS in patients with RRMM. Thus, the addition of daratumumab to subsequent treatment regimens of IMiDs (or PIs) and dexamethasone may provide an alternative salvage treatment option for patients with RRMM. Real-world data also support early medication of daratumumab to induce sustained and deep responses, and extend disorder control to potentially delay clonal evolution and subsequent drug resistance (26). However, subgroup analysis suggested that the addition of daratumumab to IMiDs (or PIs) and dexamethasone had no beneficial effect on OS. This finding warrants further validation, as the results of the subgroup analysis were obtained from only two RCTs, rendering the findings unstable and not generalizable to the entire population. Moreover, to our knowledge, several trials are still ongoing (28, 39), resulting in the absence of subgroup analysis for OS. Consequently, the subgroup analysis results concerning OS in this study await future updates. In addition, patients with RRMM who received daratumumab plus IMiDs (or PIs) and dexamethasone achieved higher VGPR or better rate than those receiving IMiDs (or PIs) and dexamethasone alone. The rates of overall response, complete response or better, and MRD-negative were also higher in the daratumumab in combination with IMiDs (or PIs) plus dexamethasone group than in the IMiDs (or PIs) plus dexamethasone group. Previous study has shown that the primary and acquired drug resistance of daratumumab is associated with tumor-related characteristics (60). It has been demonstrated that the therapeutic efficacy of daratumumab depends in part on baseline expression of CD38 on MM cells (61). Nevertheless, the effect of CD38 in acquired drug resistance remains uncertain, as CD38 is rapidly and consistently downregulated on MM cells in both responding and non-responding patients upon initiation of daratumumab therapy (62, 63).

Infections were one of the most common AEs among RRMM patients receiving daratumumab plus IMiDs (or PIs) and dexamethasone or IMiDs (or PIs) and dexamethasone, including viral infections and respiratory tract infections (41). Our meta-analysis demonstrated that the rates of URTI, pneumonia, bronchitis, and pyrexia were higher in the daratumumab in combination with IMiDs (or PIs) plus dexamethasone group than IMiDs (or PIs) and dexamethasone alone. The respiratory tract AEs may be explained by the expression of CD38 in airway muscle cells (11). Administration of antipyretics, steroids, and antihistamines is clinically suggested for standard premedication of daratumumab to prevent serious respiratory AEs (64). The updated consensus guidelines recommend considering prevention and antiviral or antibacterial vaccination, together with other means to reduce the infection risk in patients with MM (65). Moreover, RRMM patients receiving daratumumab plus IMiDs (or PIs) plus dexamethasone achieved higher rates of thrombocytopenia, neutropenia, diarrhea, back pain, fatigue, and insomnia than those receiving IMiDs (or PIs) and dexamethasone alone. It is worth noting that these TEAEs did not result in higher treatment interruption rates or fatal AEs, revealing that these hematologic and nonhematologic TEAEs were manageable. It is essential to carefully monitor patients and combine prophylaxis according to clinical assessment to manage these potential TEAEs (39).

Combined medication, including isatuximab as a backbone, can effectively treat MM. The FDA has approved combination of isatuximab with pomalidomide plus dexamethasone for the therapy of patients with MM who have received ≥ 2 previous treatments (66) or with carfilzomib and dexamethasone, for the therapy of MM patients who have received 1 to 3 prior lines of treatments (67). The pooled results of isatuximab in present study suggested that isatuximab plus IMiDs (or PIs) and dexamethasone improved PFS and OS in RRMM patients, and achieved higher rates of neutropenia, URTI, bronchitis, dyspnea, and diarrhea than IMiDs (or PIs) and dexamethasone alone. Only two studies included in this meta-analysis reported the efficacy and safety of isatuximab plus pomalidomide and dexamethasone, and isatuximab plus carfilzomib and dexamethasone, respectively. The benefits of PFS and OS can be obtained with a favorable AEs profile. Addition of isatuximab to pomalidomide increased anti-MM activity because of the direct toxicity and lysis of effector cells to tumor plasma cells. Moreover, the combination of isatuximab-PIs (i.e., carfilzomib) affects MM and microenvironmental cells (20). Given that only two studies were included in the meta-analysis of isatuximab, the pooled results of isatuximab are unstable and not convincing, and more studies need to be included to further improve the results.

There were some important limitations in our work that should be acknowledged. First, all included RCTs were the open-label design, and the potential bias due to lack of blinding may affect the outcomes. Second, the TEAEs caused by anti-CD38 mAbs cannot be ignored, and the present study did not explore in detail whether these TEAEs are associated with IMiDs (or PIs) and dexamethasone. Third, TSA results suggested that the sample size for the TEAEs, such as anemia, thrombocytopenia, lymphopenia, constipation, arthralgia, asthenia and hypertension, did not reach the required sample size to achieve a definitive conclusion. Therefore, future inclusion of studies with large sample size is needed to further let the results of these TEAEs more reliable.

## 5 Conclusion

Taken together, our meta-analysis demonstrated that anti-CD38 mAbs in combination with IMiDs (or PIs) and dexamethasone improved PFS and OS in patients with RRMM, and achieved higher rates of overall response, complete response or better, VGPR or better, and MRD-negative compared with IMiDs (or PIs) and dexamethasone alone. The rates of hematologic and nonhematologic TEAEs, including thrombocytopenia, neutropenia, URTI, pneumonia, bronchitis, dyspnea, diarrhea, pyrexia, back pain, arthralgia, fatigue, insomnia, and hypertension, were also higher in the anti-CD38 mAbs in combination with IMiDs (or PIs) and dexamethasone group than in the IMiDs (or PIs) and dexamethasone group.

## Data availability statement

The original contributions presented in the study are included in the article/**Supplementary Material**. Further inquiries can be directed to the corresponding authors.

## Author contributions

LZo, NH, and LYa participated in research design. YX, QY, RJ, and LZ searched the literature, selected studies for inclusion, and collected the data. DC and MX analyzed the data. LYe and FZ prepared the manuscript. LZh, NH, and XY edited the manuscript. All authors contributed to the article and approved the submitted version.
