# Supplementary material for: Efficacy and safety of anti-CD38 monoclonal antibodies in patients with relapsed/refractory multiple myeloma: a systematic review and meta-analysis with trial sequential analysis of randomized controlled trials
Source: Front Oncol. 2023 Dec 7;13:1240318. doi: 10.3389/fonc.2023.1240318 (PMC10746851; doi:10.3389/fonc.2023.1240318)
Supplement: Supplementary file 1 [file DataSheet_1.docx]

**PubMed** 173

#1 daratumumab OR humax-CD38 OR Darzalex

#2 isatuximab OR Sarclisa OR SAR650984 OR isatuximab-irfc OR SAR

#3 (multiple myeloma) OR (multiple myelomas) OR (plasma cell myeloma) OR (plasma cell myelomas) OR myelomatosis OR myelomatoses OR (kahler disease) OR (myeloma-multiple) OR (myeloma-multiples)

#4 (randomized controlled trial) OR (randomised controlled trial) OR (controlled clinical trial) OR randomized OR placebo

#5 (#1 OR #2) AND #3 AND #4

**Web of Science** 293

#1 TS=(daratumumab OR humax-CD38 OR Darzalex)

#2 TS=(isatuximab OR Sarclisa OR SAR650984 OR isatuximab-irfc OR SAR)

#3 TS=((multiple myeloma) OR (multiple myelomas) OR (plasma cell myeloma) OR (plasma cell myelomas) OR myelomatosis OR myelomatoses OR (kahler disease) OR (myeloma-multiple) OR (myeloma-multiples))

#4 (randomized controlled trial) OR (randomised controlled trial) OR (controlled clinical trial) OR randomized OR placebo

#5 (#1 OR #2) AND #3 AND #4

**Embase** 1557

#1 'daratumumab'/exp OR 'humax cd38'/exp OR 'darzalex'/exp

#2 'isatuximab'/exp OR 'sarclisa'/exp OR 'sar650984'/exp OR 'isatuximab irfc'/exp OR 'sar'/exp

#3 'multiple myeloma'/exp OR 'multiple myelomas' OR 'plasma cell myeloma'/exp OR 'plasma cell myelomas' OR 'myelomatosis'/exp OR myelomatoses OR 'kahler disease'/exp OR 'myeloma multiple'/exp OR 'myeloma multiples'

#4 randomized AND controlled AND trial OR (randomised AND controlled AND trial) OR (controlled AND clinical AND trial) OR randomized OR placebo

#5 (#1 OR #2) AND #3 AND #4

**The Cochrane Library** 643

#1 daratumumab OR humax-CD38 OR Darzalex

#2 isatuximab OR Sarclisa OR SAR650984 OR isatuximab-irfc OR SAR

#3 (multiple myeloma) OR (multiple myelomas) OR (plasma cell myeloma) OR (plasma cell myelomas) OR myelomatosis OR myelomatoses OR (kahler disease) OR (myeloma-multiple) OR (myeloma-multiples)

#4 (randomized controlled trial) OR (randomised controlled trial) OR (controlled clinical trial) OR randomized OR placebo

#5 (#1 OR #2) AND #3 AND #4
