# Supplementary material for: Efficacy and safety of anti-CD38 monoclonal antibodies in patients with relapsed/refractory multiple myeloma: a systematic review and meta-analysis with trial sequential analysis of randomized controlled trials
Source: Front Oncol. 2023 Dec 7;13:1240318. doi: 10.3389/fonc.2023.1240318 (PMC10746851; doi:10.3389/fonc.2023.1240318)
Supplement: Supplementary file 2 [file DataSheet_2.docx]

| TABLE S1 Quality analysis of the included studies by modified Jadad scale. | | | | | | |
| --- | --- | --- | --- | --- | --- | --- |
| Study | Randomization | Randomization concealment | Double blind | Withdrawals and dropouts | Score | Study quality |
| Richardson (2022) | 2 | 2 | 0 | 1 | 5 | High |
| Usmani (2023) | 2 | 2 | 0 | 1 | 5 | High |
| Sonneveld (2023) | 2 | 2 | 0 | 1 | 5 | High |
| Lu (2021) | 1 | 0 | 0 | 0 | 1 | Low |
| Martin (2023) | 2 | 2 | 0 | 1 | 5 | High |
| Dimopoulos (2021) | 2 | 2 | 0 | 1 | 5 | High |
| Bahlis (2020) | 2 | 2 | 0 | 0 | 4 | High |
| Fu (2023) | 1 | 0 | 0 | 0 | 1 | Low |
| Usmani (2022) | 2 | 2 | 0 | 1 | 5 | High |
| Dimopoulos (2023) | 2 | 2 | 0 | 1 | 5 | High |
| Mateos (2020) | 2 | 2 | 0 | 0 | 4 | High |
