# Supplementary material for: Efficacy and safety of anti-CD38 monoclonal antibodies in patients with relapsed/refractory multiple myeloma: a systematic review and meta-analysis with trial sequential analysis of randomized controlled trials
Source: Front Oncol. 2023 Dec 7;13:1240318. doi: 10.3389/fonc.2023.1240318 (PMC10746851; doi:10.3389/fonc.2023.1240318)

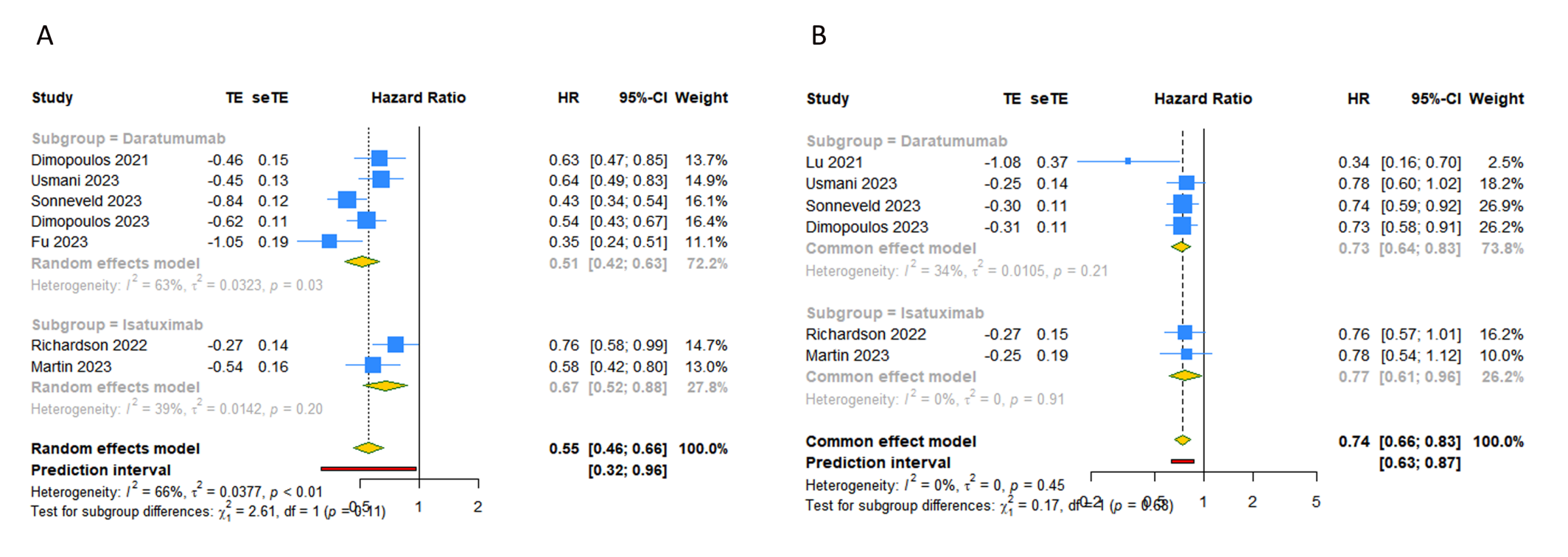


**FIGURE S1** Subgroup analysis of progression-free survival (PFS) and overall survival (OS) after anti-CD38 mAbs therapy for RRMM. (A) PFS; (B) OS.

**FIGURE S2** Subgroup analysis of the efficacy outcomes after anti-CD38 mAbs therapy for RRMM. (A) Overall response rate; (B) Complete response or better rate; (C) Very good partial response or better rate; (D) Minimum residual disease-negative rate.


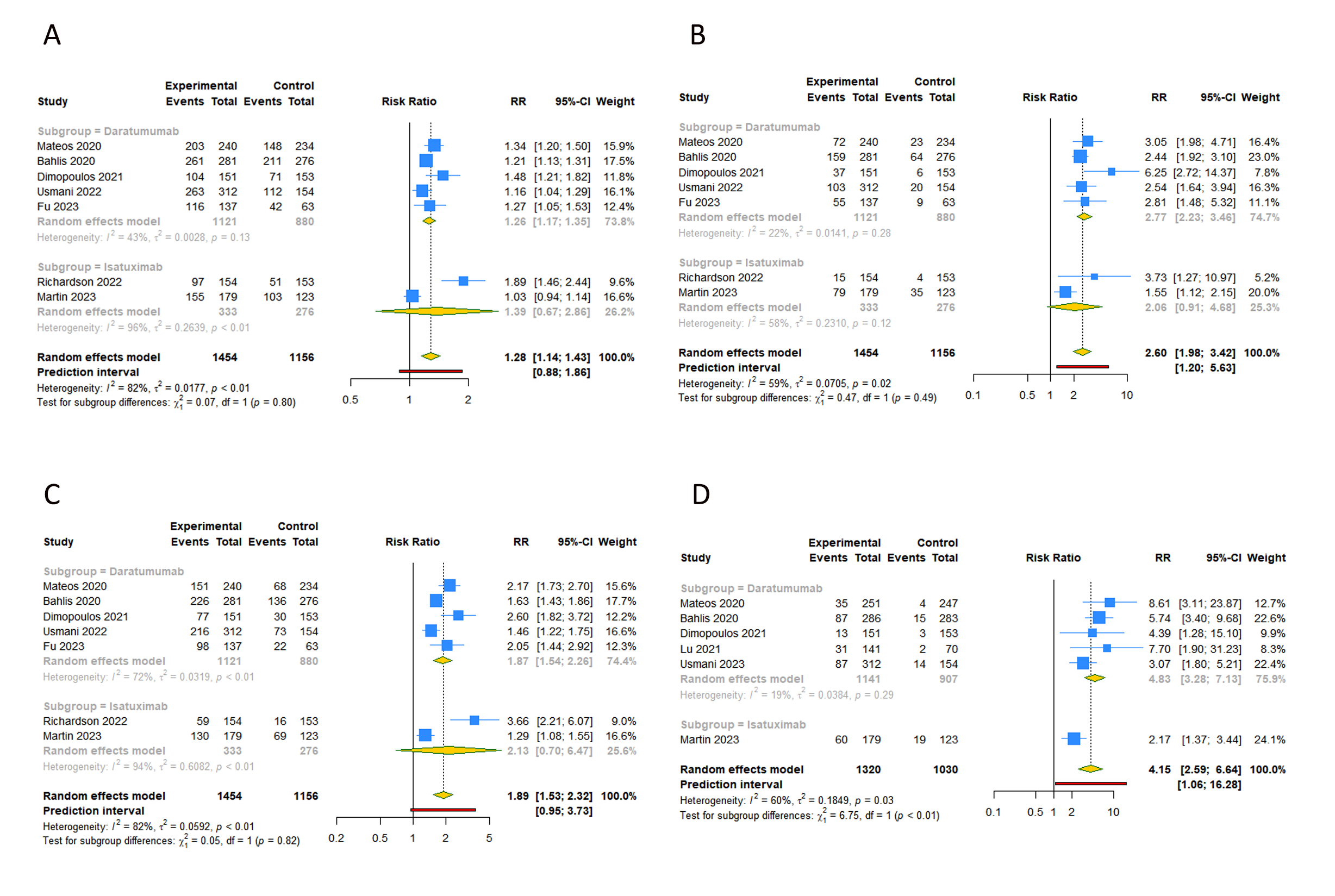


**FIGURE S3** Subgroup analysis of the hematologic treatment-emergent adverse events of anti-CD38 mAbs therapy for RRMM. (A) Anemia; (B) Thrombocytopenia; (C) Neutropenia; (D) Lymphopenia.


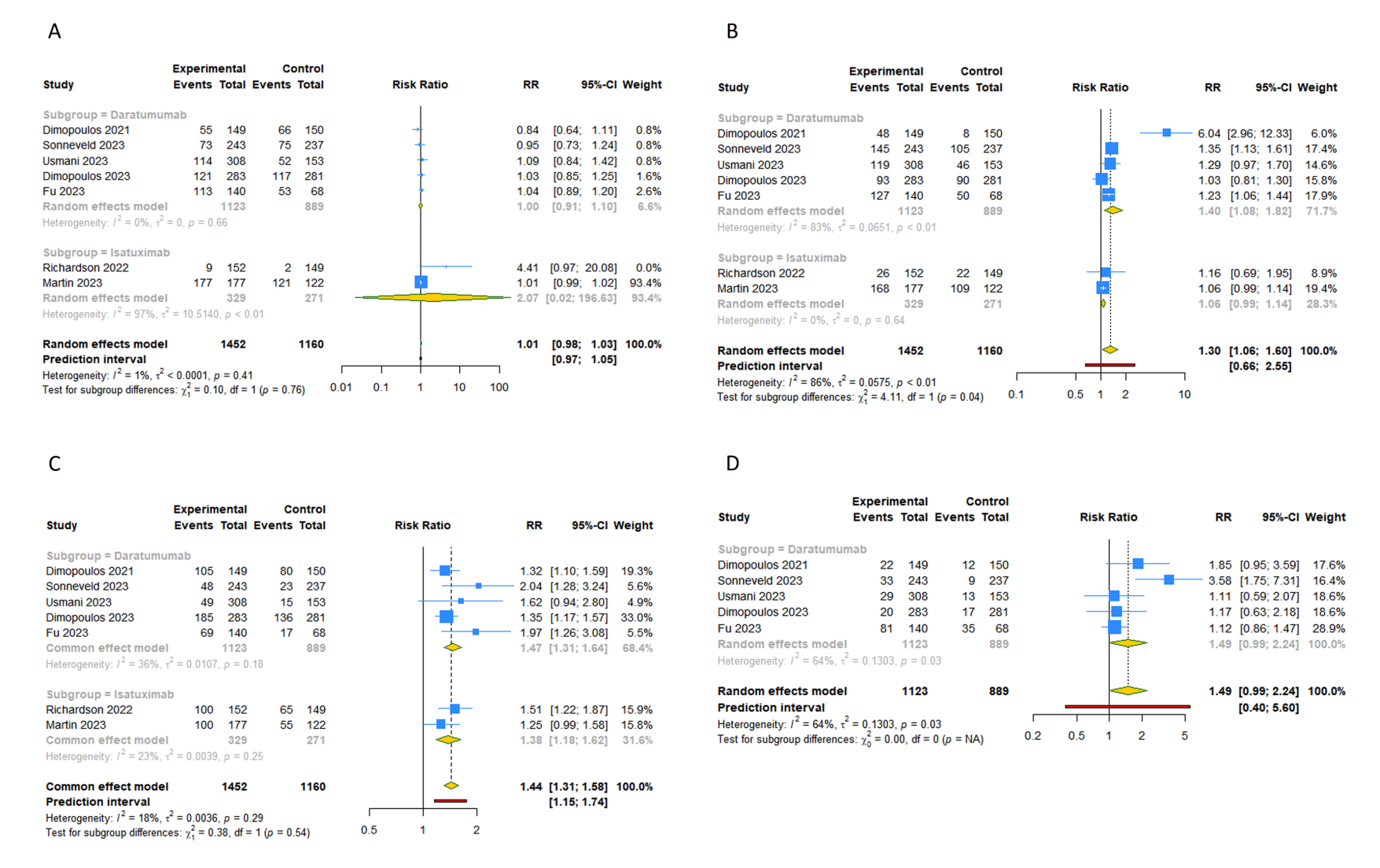


**FIGURE S4** Subgroup analysis of respiratory system treatment-emergent adverse events of anti-CD38 mAbs therapy for RRMM. (A) Upper respiratory tract infection; (B) Pneumonia; (C) Bronchitis; (D) Dyspnea.


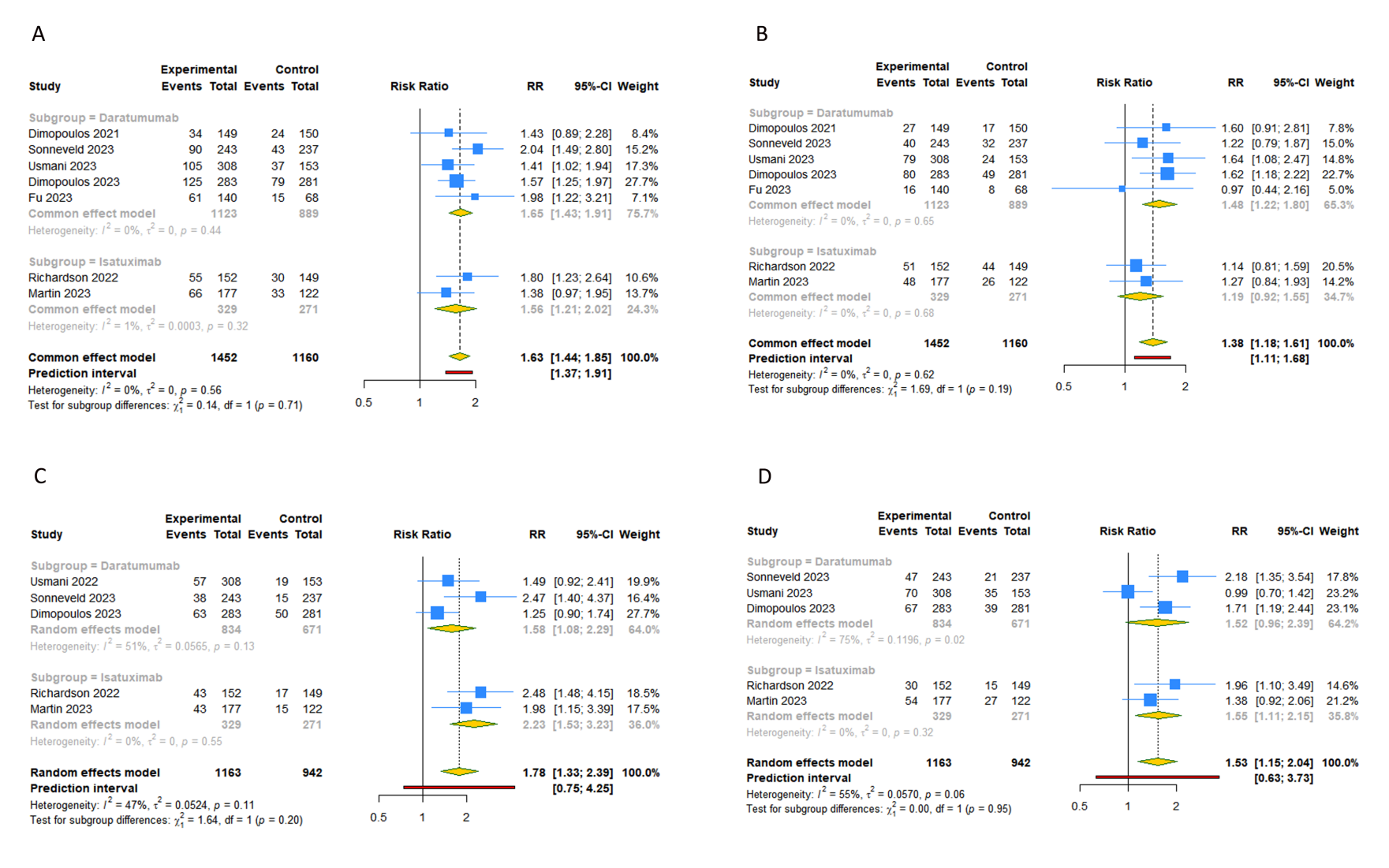


**FIGURE S5** Subgroup analysis of digestive system treatment-emergent adverse events of anti-CD38 mAbs therapy for RRMM. (A) Diarrhea; (B) Constipation.


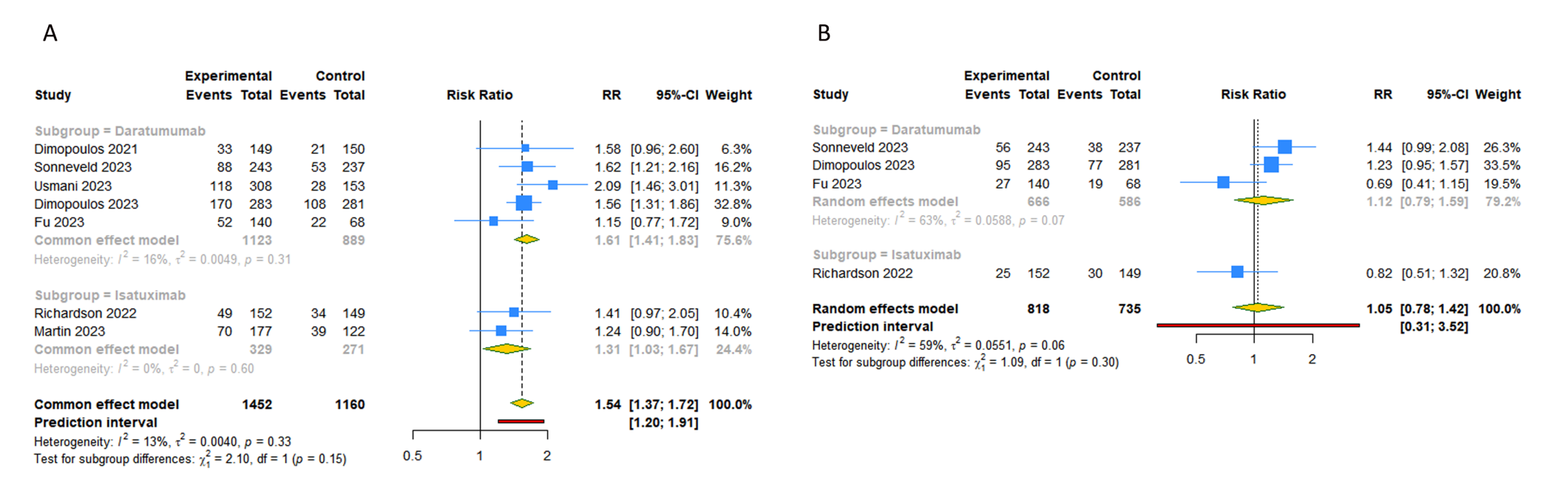


**FIGURE S6** Subgroup analysis of other nonhematologic treatment-emergent adverse events of anti-CD38 mAbs therapy for RRMM. (A) Pyrexia; (B) Back pain; (C) Arthralgia; (D) Fatigue; (E) Asthenia; (F) Insomnia; (G) Hypertension.


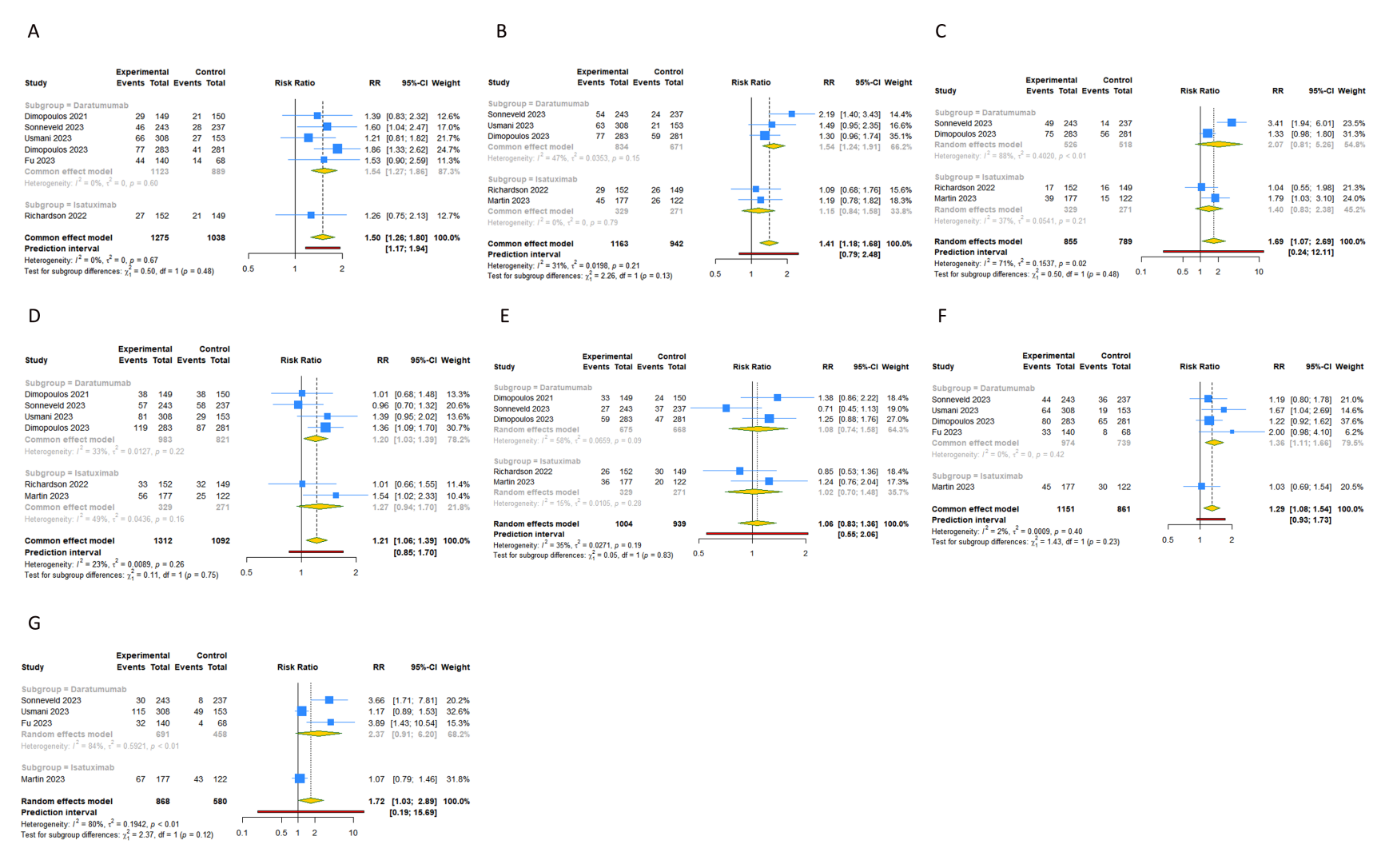

Supplement: Supplementary file 3 [file DataSheet_3.docx]
