# Supplementary material for: Efficacy and safety of anti-CD38 monoclonal antibodies in patients with relapsed/refractory multiple myeloma: a systematic review and meta-analysis with trial sequential analysis of randomized controlled trials
Source: Front Oncol. 2023 Dec 7;13:1240318. doi: 10.3389/fonc.2023.1240318 (PMC10746851; doi:10.3389/fonc.2023.1240318)

| **TABLE S1** Subgroup analysis of PFS after Daratumumab treatment for RRMM. | | | | | | | |
| --- | --- | --- | --- | --- | --- | --- | --- |
| Groups | Number of study | Meta-analysis | | | | Heterogeneity | |
|  |  | HR | 95% CI | P value | 95% PI | I^2^, Tau^2^ | P value |
| Sex | | | | | | | |
| Male | 2 | 0.530 | 0.311-0.904 | 0.020 | - | 70.0%, 0.1040 | 0.068 |
| Female | 2 | 0.470 | 0.335-0.659 | <0.001 |  | 10.5%, 0.0075 | 0.291 |
| Age | | | | | | | |
| < 65 years | 5 | 0.461 | 0.389-0.547 | <0.001 | 0.310-0.694 | 15.5%, 0.0070 | 0.316 |
| ≥ 65 years | 5 | 0.436 | 0.308-0.617 | <0.001 | 0.130-1.463 | 74.2%, 0.1135 | 0.004 |
| International Staging System disease staging | | | | | | | |
| I | 4 | 0.368 | 0.257-0.528 | <0.001 | 0.082-1.649 | 65.3%, 0.0877 | 0.034 |
| II | 4 | 0.411 | 0.330-0.511 | <0.001 | 0.205-0.843 | 18.0%, 0.0114 | 0.301 |
| III | 5 | 0.532 | 0.414-0.683 | <0.001 | 0.354-0.799 | 0%, 0 | 0.672 |
| Type of measurable MM | | | | | | | |
| IgG | 4 | 0.421 | 0.292-0.605 | <0.001 | 0.089-1.994 | 71.0%, 0.0964 | 0.016 |
| Non-IgG | 4 | 0.428 | 0.322-0.569 | <0.001 | 0.229-0.799 | 0%, 0 | 0.806 |
| Number of prior lines of therapy | | | | | | | |
| 1 | 5 | 0.423 | 0.275-0.652 | <0.001 | 0.095-1.882 | 74.9%, 0.1713 | 0.003 |
| 2 | 3 | 0.418 | 0.321-0.545 | <0.001 | 0.075-2.324 | 0%, 0 | 0.860 |
| 3 | 3 | 0.545 | 0.365-0.813 | 0.003 | 0.041-7.308 | 0%, 0 | 0.885 |
| >3 | 3 | 0.365 | 0.227-0.589 | <0.001 | 0.009-14.975 | 9.0%, 0.0187 | 0.333 |
| Cytogenetic profile | | | | | | | |
| High risk | 5 | 0.502 | 0.380-0.662 | <0.001 | 0.216-1.127 | 27.7%, 0.0390 | 0.237 |
| Standard risk | 5 | 0.397 | 0.304-0.517 | <0.001 | 0.173-0.907 | 55.3%, 0.0492 | 0.063 |

| **TABLE S2** Subgroup analysis of OS after Daratumumab treatment for RRMM. | | | | | | | |
| --- | --- | --- | --- | --- | --- | --- | --- |
| Groups | Number of study | Meta-analysis | | | | Heterogeneity | |
|  |  | HR | 95% CI | P value | 95% PI | I^2^, Tau^2^ | P value |
| Sex | | | | | | | |
| Male | 2 | 0.789 | 0.645-0.966 | 0.022 | - | 0%, 0 | 0.424 |
| Female | 2 | 0.696 | 0.539-0.898 | 0.005 | - | 0%, 0 | 0.583 |
| Age | | | | | | | |
| < 65 years | 3 | 0.738 | 0.604-0.903 | 0.003 | 0.200-2.724 | 0%, 0 | 0.597 |
| ≥ 65 years | 3 | 0.785 | 0.648-0.950 | 0.013 | 0.165-3.734 | 12.1%, 0.0041 | 0.321 |
| International Staging System disease staging | | | | | | | |
| I | 2 | 0.732 | 0.563-0.953 | 0.021 | - | 0%, 0 | 0.761 |
| II | 2 | 0.719 | 0.557-0.929 | 0.011 | - | 0%, 0 | 0.915 |
| III | 3 | 0.703 | 0.533-0.927 | 0.013 | - | 0%, 0 | 0.716 |
| Type of measurable MM | | | | | | | |
| IgG | 2 | 0.752 | 0.601-0.942 | 0.013 | - | 0%, 0 | 0.525 |
| Non-IgG | 2 | 0.814 | 0.579-1.145 | 0.237 | - | 0%, 0 | 0.724 |
| Number of prior lines of therapy | | | | | | | |
| 1 | 3 | 0.681 | 0.555-0.836 | <0.001 | 0.180-2.577 | 0%, 0 | 0.429 |
| 2 | 2 | 0.805 | 0.603-1.075 | 0.141 | - | 0%, 0 | 0.615 |
| 3 | 2 | 0.904 | 0.593-1.376 | 0.637 | - | 0%, 0 | 0.335 |
| >3 | 2 | 1.058 | 0.605-1.849 | 0.844 | - | 0%, 0 | 0.836 |
| Cytogenetic profile | | | | | | | |
| High risk | 3 | 0.654 | 0.467-0.916 | 0.013 | 0.073-5.814 | 0%, 0 | 0.644 |
| Standard risk | 3 | 0.737 | 0.610-0.892 | 0.002 | 0.216-2.524 | 0%, 0 | 0.644 |


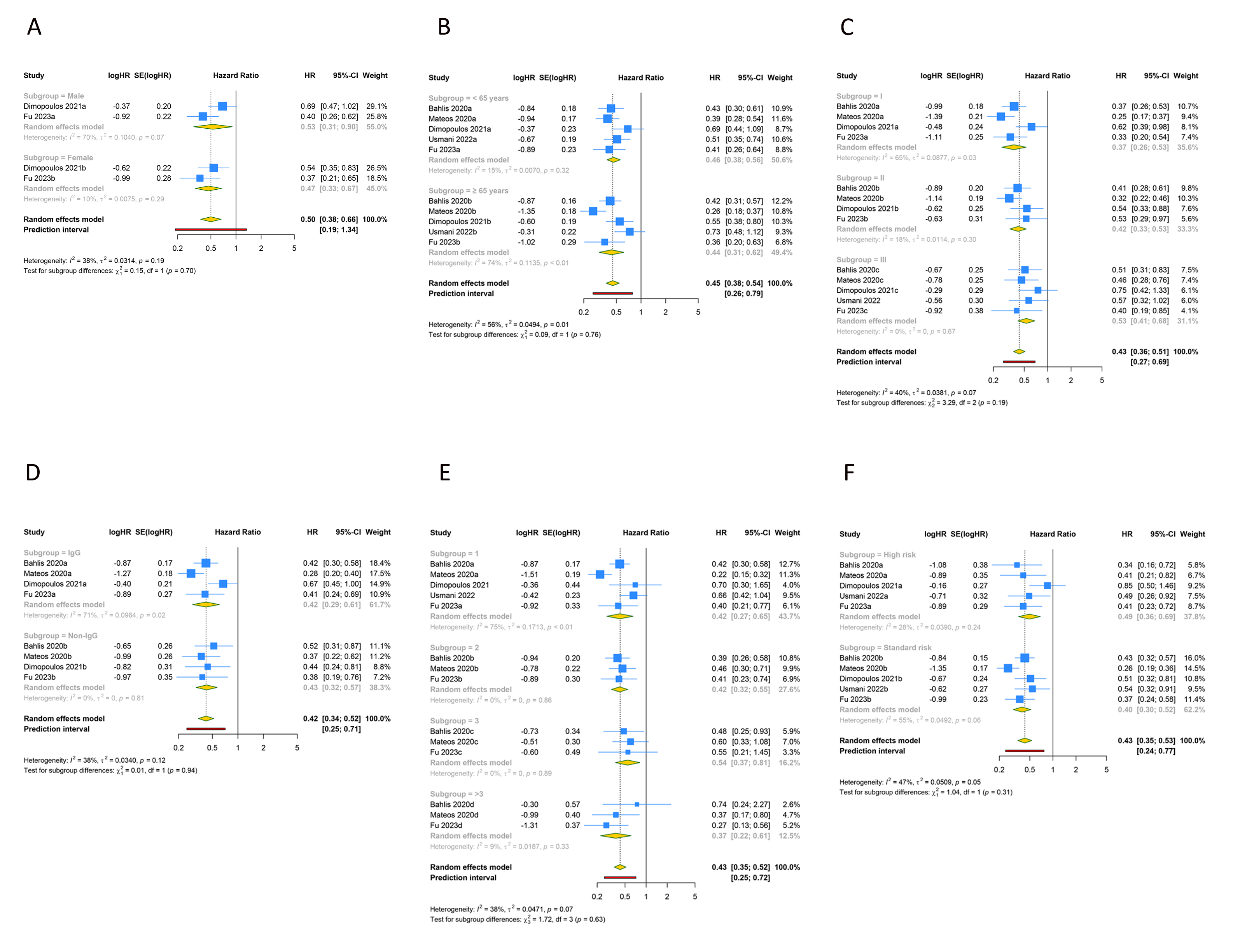


**FIGURE S1** Subgroup analysis of progression-free survival (PFS) after Daratumumab therapy for RRMM. A-F: Subgrouped by sex (A), age (B), International Staging System disease staging (C), type of measurable multiple myeloma (D), number of prior lines of therapy (E), and cytogenetic profile (F).

**FIGURE S2** Subgroup analysis of overall survival (OS) after Daratumumab therapy for RRMM. A-F: Subgrouped by sex (A), age (B), International Staging System disease staging (C), type of measurable multiple myeloma (D), number of prior lines of therapy (E), and cytogenetic profile (F).


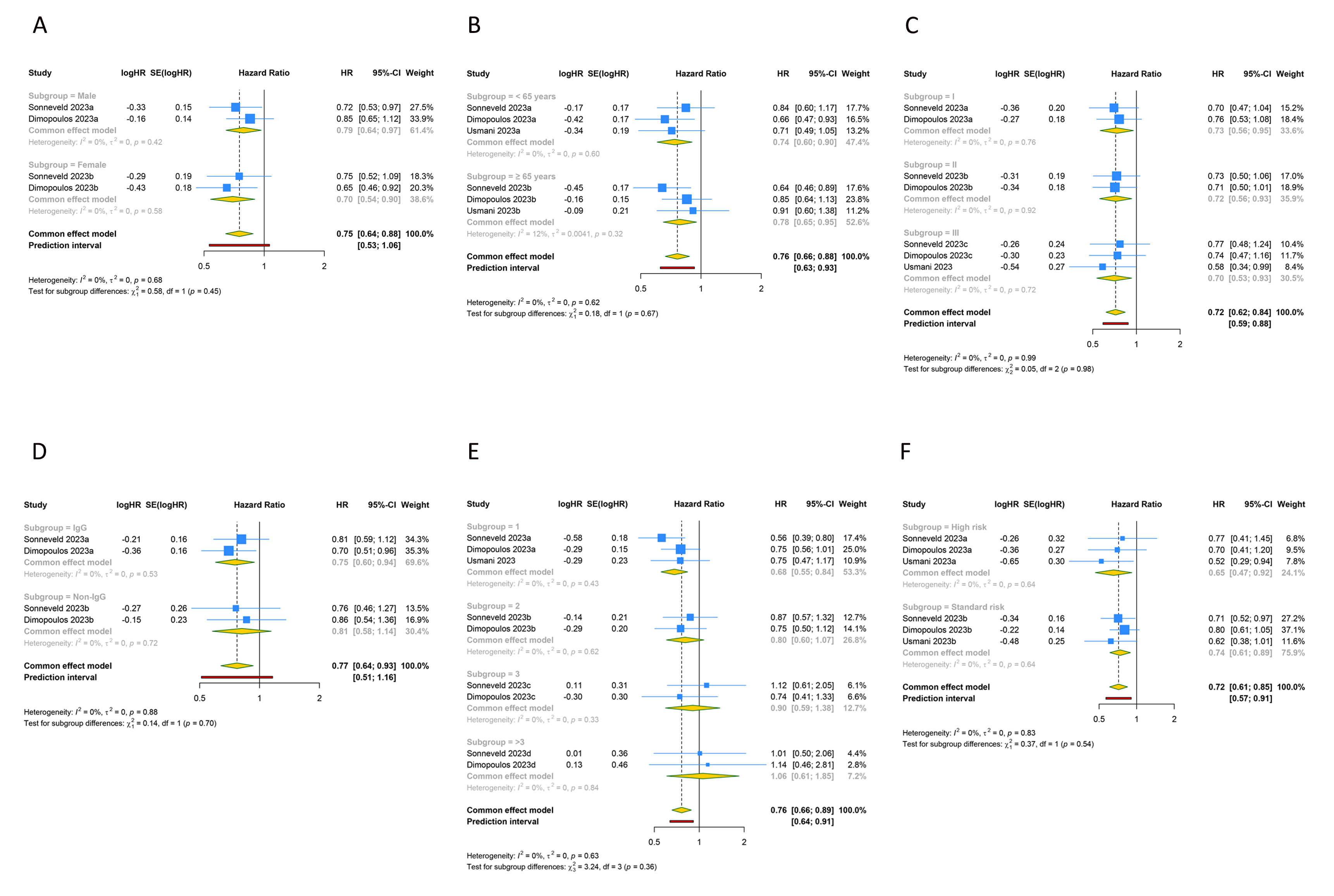

Supplement: Supplementary file 4 [file DataSheet_4.docx]
