# Supplementary material for: Efficacy and safety of anti-CD38 monoclonal antibodies in patients with relapsed/refractory multiple myeloma: a systematic review and meta-analysis with trial sequential analysis of randomized controlled trials
Source: Front Oncol. 2023 Dec 7;13:1240318. doi: 10.3389/fonc.2023.1240318 (PMC10746851; doi:10.3389/fonc.2023.1240318)

**FIGURE S1** Trial sequential analysis (TSA) of the efficacy outcomes after anti-CD38 mAbs therapy for RRMM. (A) Overall response rate; (B) Complete response or better rate; (C) Very good partial response or better rate; (D) Minimum residual disease-negative rate. Uppermost and lowermost red curves represent trial sequential monitoring boundary lines for benefit and harm, respectively. Horizontal green lines represent the conventional boundaries for statistical significance. Inner red lines represent the futility boundary.


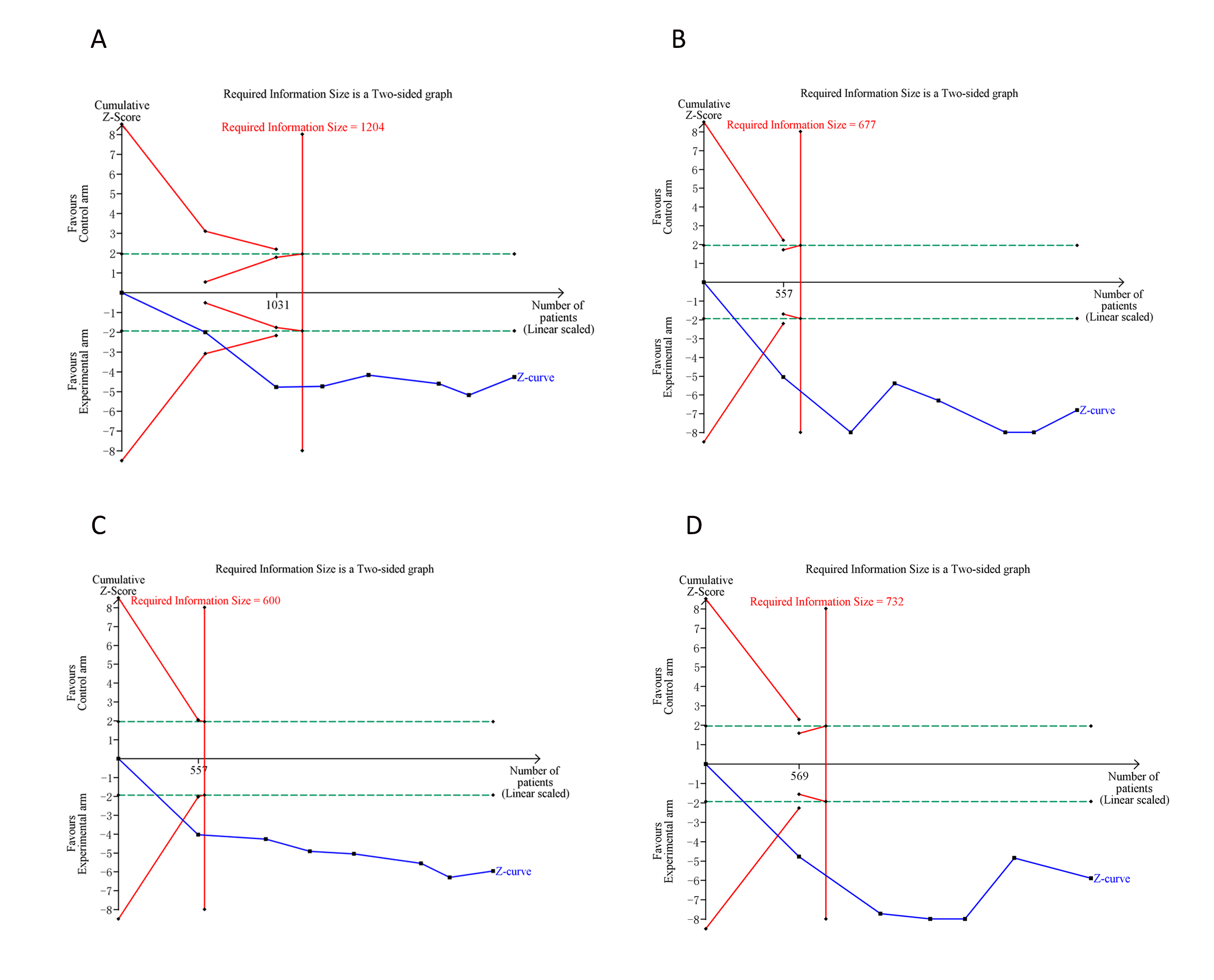


**FIGURE S2** Trial sequential analysis (TSA) of the hematologic treatment-emergent adverse events of anti-CD38 mAbs therapy for RRMM. (A) Anemia; (B) Thrombocytopenia; (C) Neutropenia; (D) Lymphopenia. Uppermost and lowermost red curves represent trial sequential monitoring boundary lines for benefit and harm, respectively. Horizontal green lines represent the conventional boundaries for statistical significance. Inner red lines represent the futility boundary.


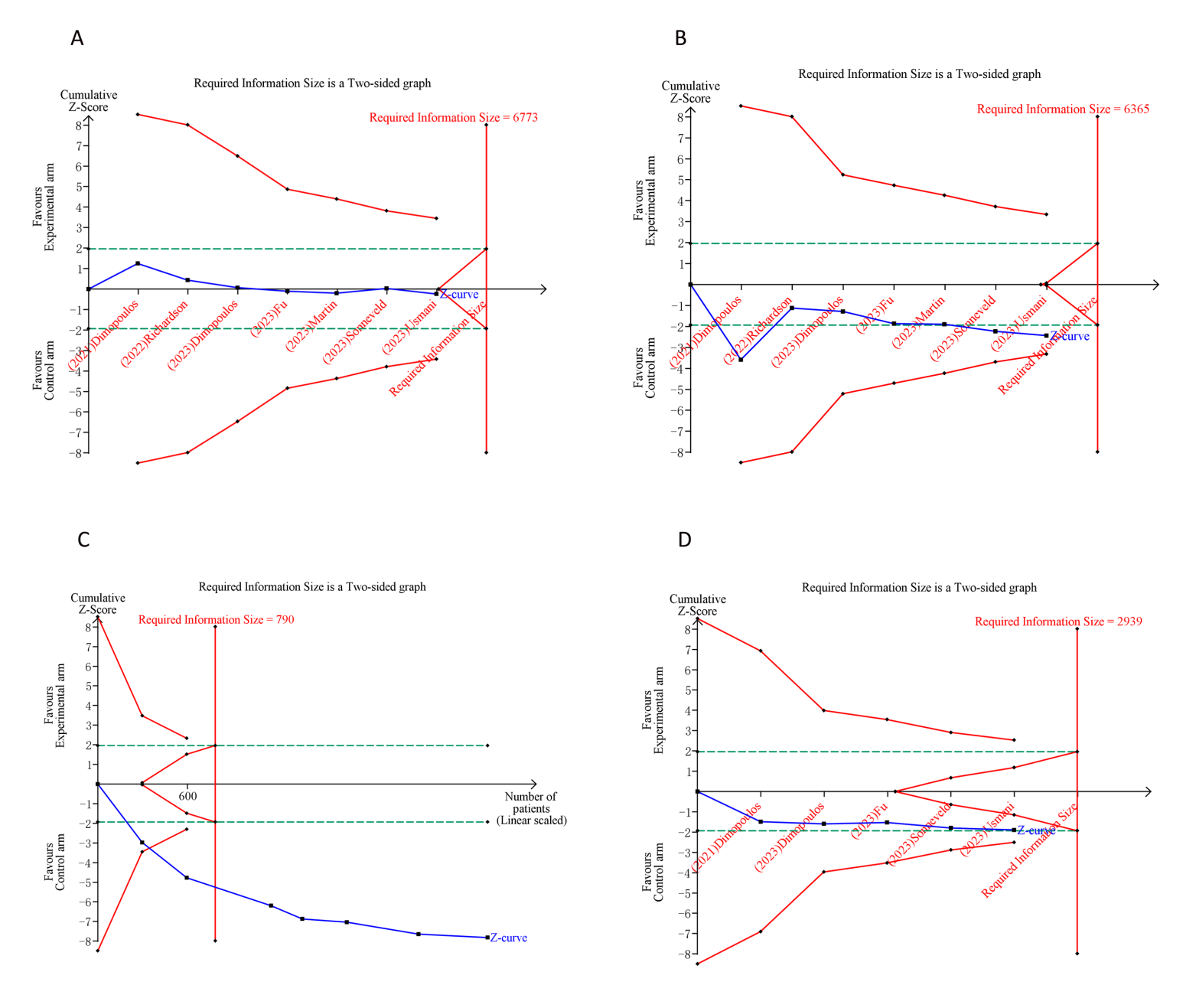


**FIGURE S3** Trial sequential analysis (TSA) of respiratory system treatment-emergent adverse events of anti-CD38 mAbs therapy for RRMM. (A) Upper respiratory tract infection; (B) Pneumonia; (C) Bronchitis; (D) Dyspnea. Uppermost and lowermost red curves represent trial sequential monitoring boundary lines for benefit and harm, respectively. Horizontal green lines represent the conventional boundaries for statistical significance. Inner red lines represent the futility boundary.


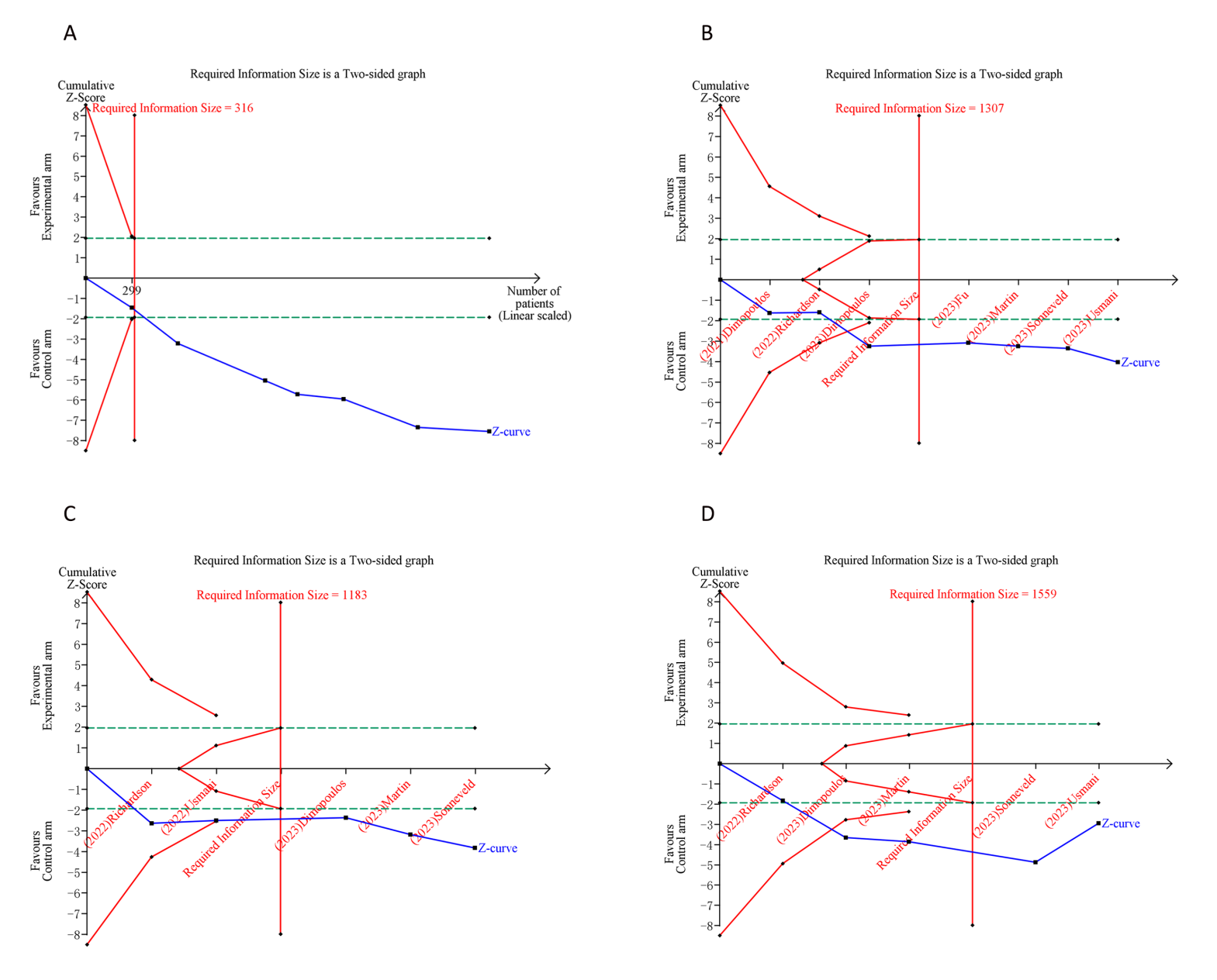


**FIGURE S4** Trial sequential analysis (TSA) of digestive system treatment-emergent adverse events of anti-CD38 mAbs therapy for RRMM. (A) Diarrhea; (B) Constipation. Uppermost and lowermost red curves represent trial sequential monitoring boundary lines for benefit and harm, respectively. Horizontal green lines represent the conventional boundaries for statistical significance. Inner red lines represent the futility boundary.


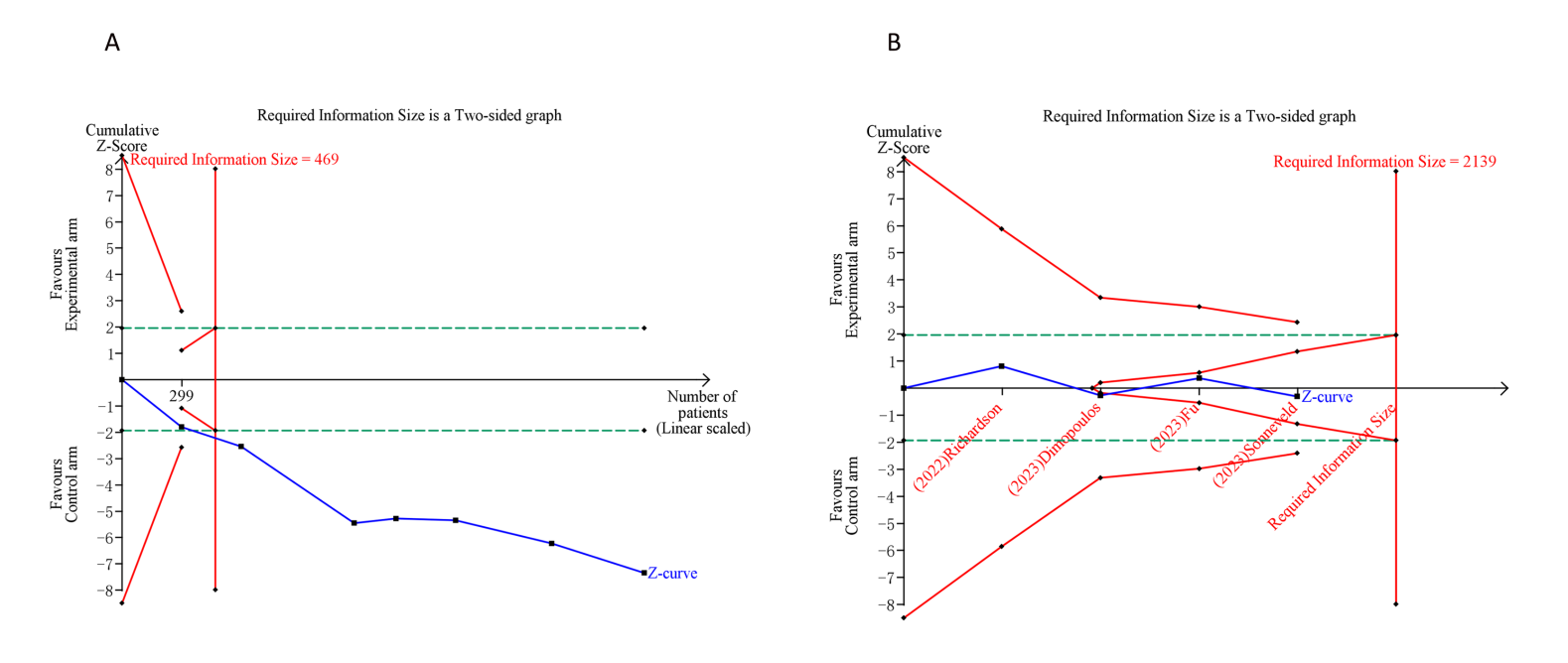


**FIGURE S5** Trial sequential analysis (TSA) of other nonhematologic treatment-emergent adverse events of anti-CD38 mAbs therapy for RRMM. (A) Pyrexia; (B) Back pain; (C) Arthralgia; (D) Fatigue; (E) Asthenia; (F) Insomnia; (G) Hypertension. Uppermost and lowermost red curves represent trial sequential monitoring boundary lines for benefit and harm, respectively. Horizontal green lines represent the conventional boundaries for statistical significance. Inner red lines represent the futility boundary.


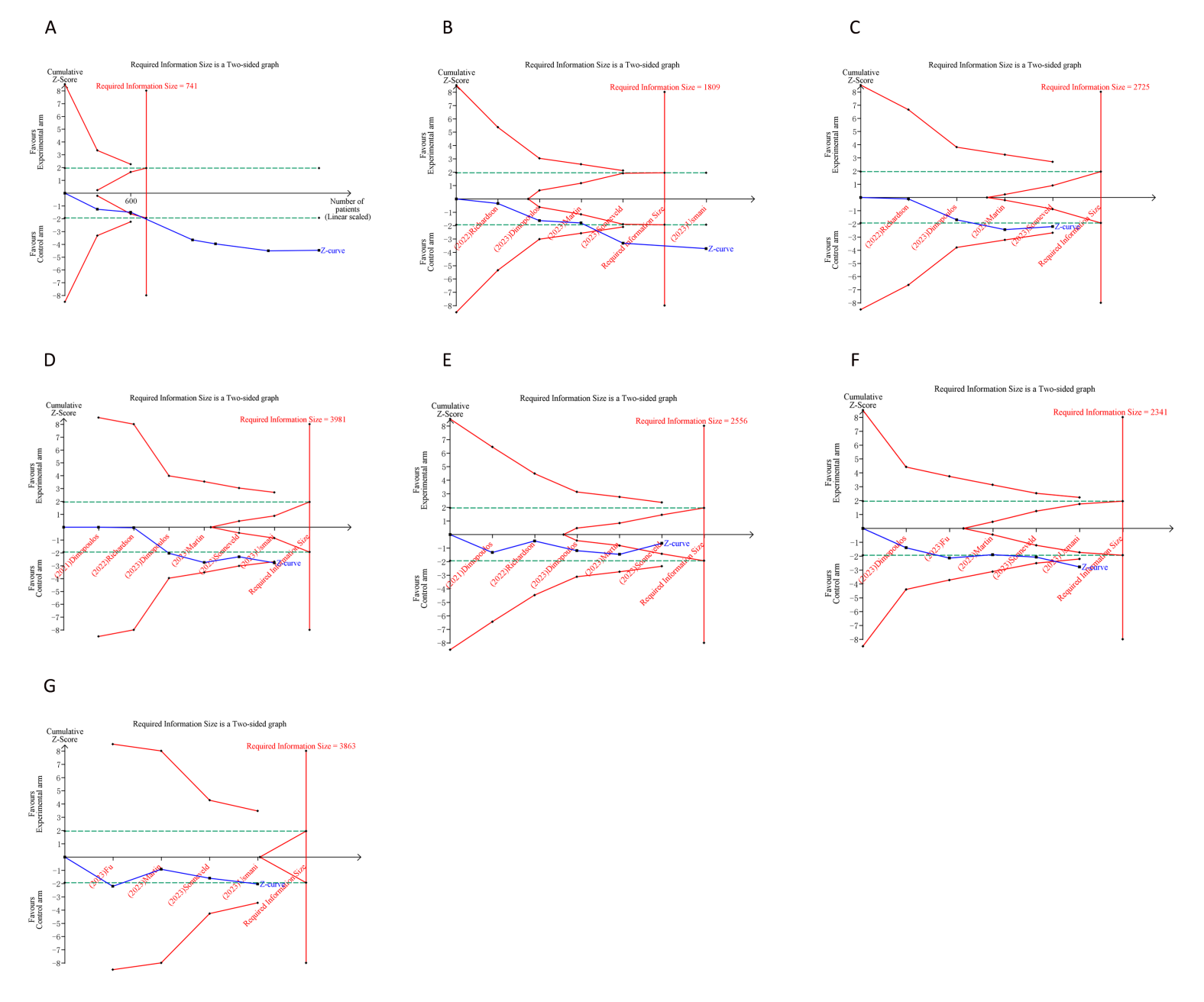

Supplement: Supplementary file 5 [file DataSheet_5.docx]
