# Supplementary material for: Efficacy and safety of anti-CD38 monoclonal antibodies in patients with relapsed/refractory multiple myeloma: a systematic review and meta-analysis with trial sequential analysis of randomized controlled trials
Source: Front Oncol. 2023 Dec 7;13:1240318. doi: 10.3389/fonc.2023.1240318 (PMC10746851; doi:10.3389/fonc.2023.1240318)

**FIGURE S1** Funnel plot of trim-and-fill method on the efficacy outcomes after anti-CD38 mAbs therapy for RRMM. (A) Progression-free survival; (B) Overall response rate; (C) Complete response or better rate; (D) Very good partial response or better rate.


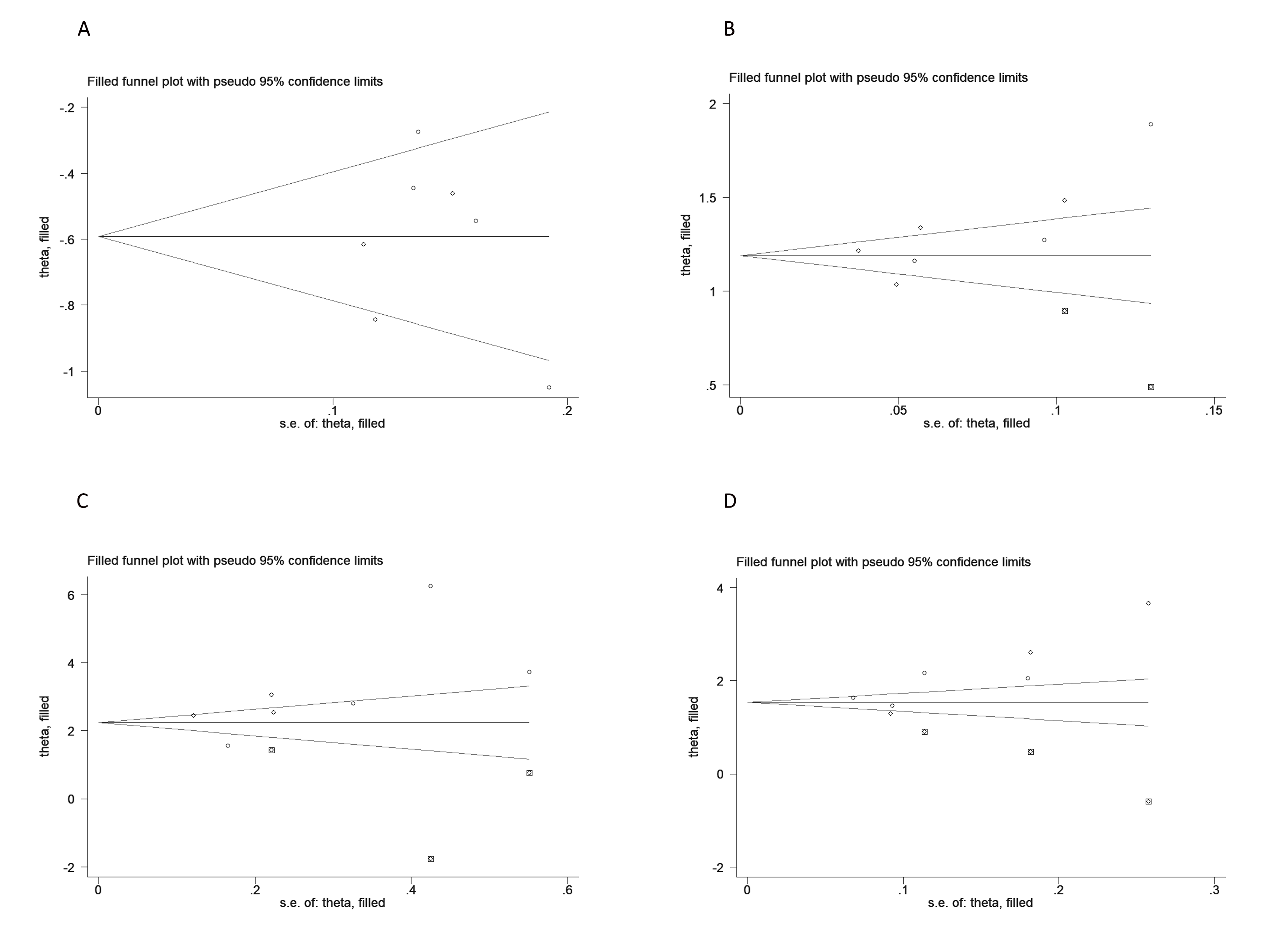


**FIGURE S2** Funnel plot of trim-and-fill method on the hematologic treatment-emergent adverse events of anti-CD38 mAbs therapy for RRMM. (A) Anemia; (B) Thrombocytopenia; (C) Neutropenia.


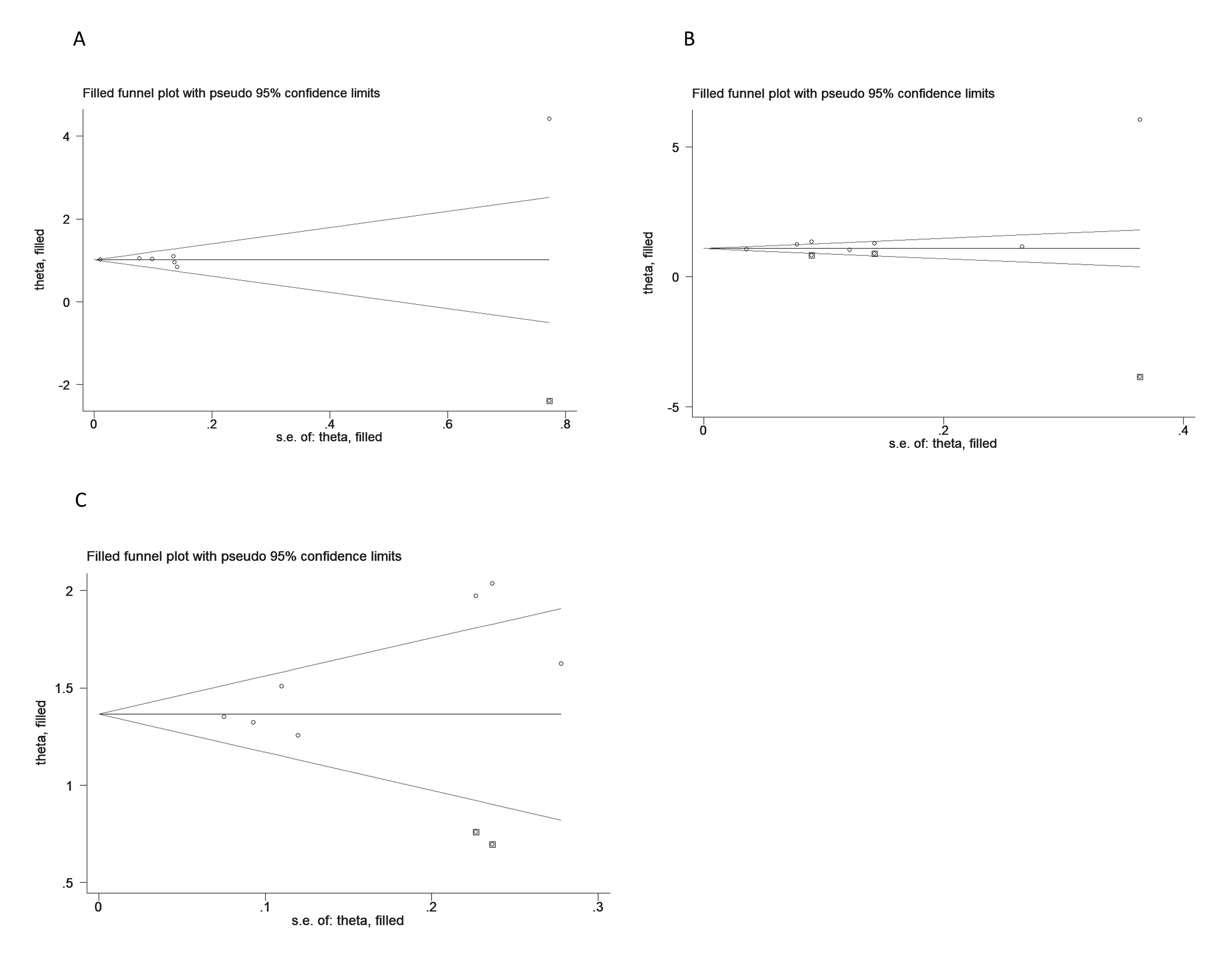


**FIGURE S3** Funnel plot of trim-and-fill method on the nonhematologic treatment-emergent adverse events of anti-CD38 mAbs therapy for RRMM. (A) Upper respiratory tract infection; (B) Pneumonia; (C) Diarrhea.

**FIGURE S4** Sensitivity analysis on the efficacy outcomes after anti-CD38 mAbs therapy for RRMM. (A) Progression-free survival; (B) Overall response rate; (C) Complete response or better rate; (D) Very good partial response or better rate.


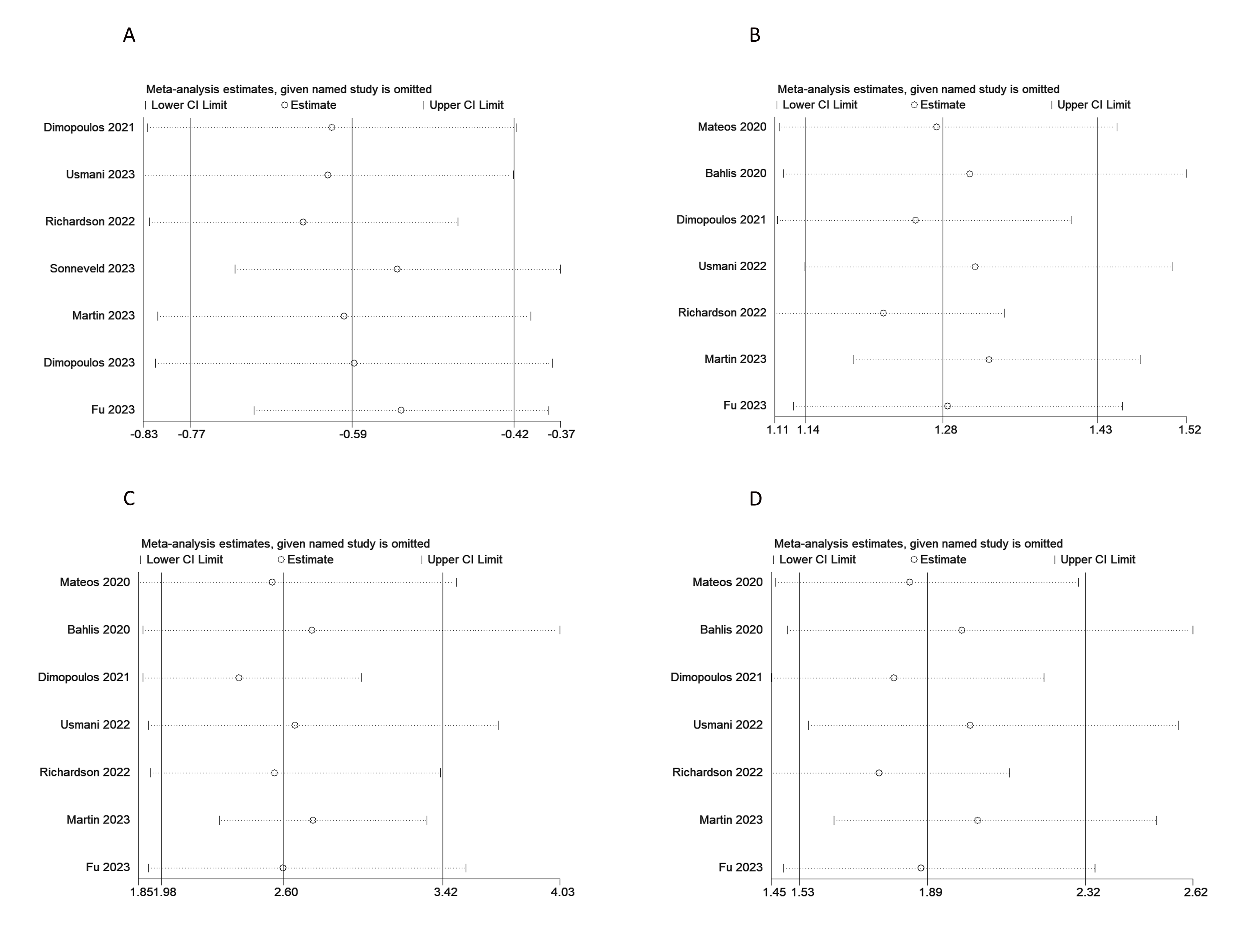


**FIGURE S5** Sensitivity analysis on the hematologic treatment-emergent adverse events of anti-CD38 mAbs therapy for RRMM. (A) Anemia; (B) Thrombocytopenia; (C) Neutropenia.


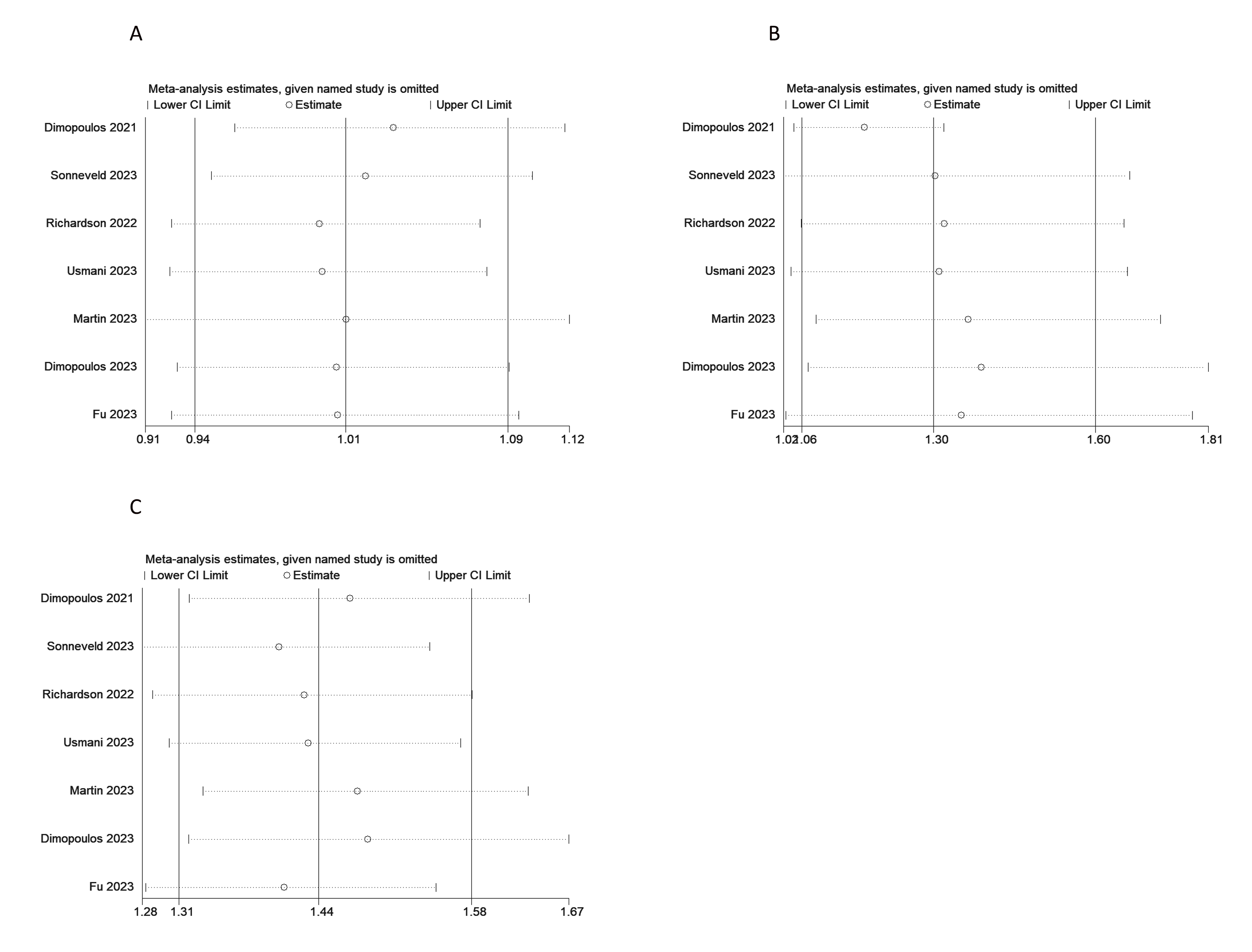


**FIGURE S6** Sensitivity analysis on the nonhematologic treatment-emergent adverse events of anti-CD38 mAbs therapy for RRMM. (A) Upper respiratory tract infection; (B) Pneumonia; (C) Diarrhea.


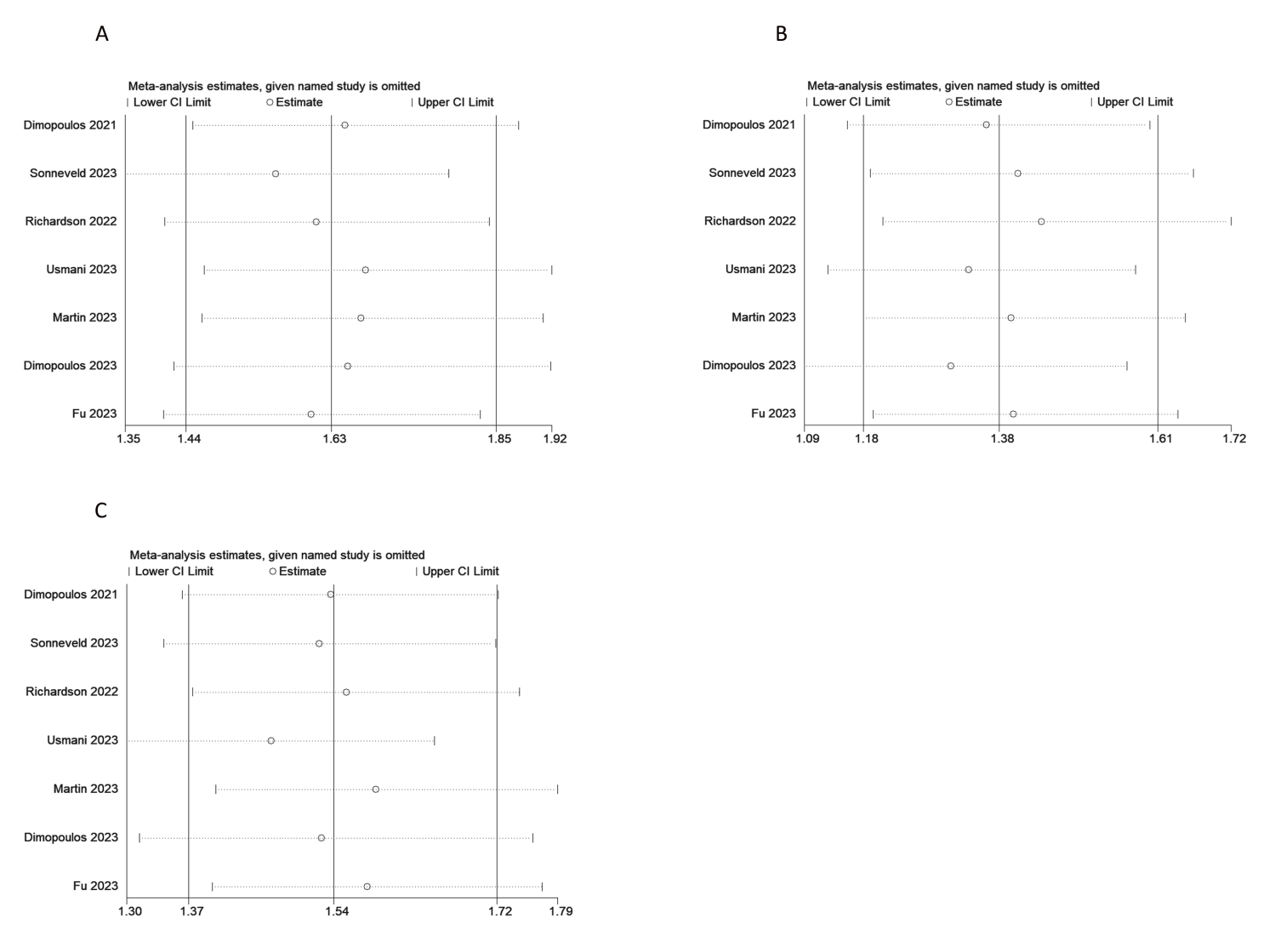

Supplement: Supplementary file 6 [file DataSheet_6.docx]
